# Knowledge, attitudes, and prevention practices regarding human papillomavirus among health and social science female students in Northwest Ethiopia: A comparative cross-sectional study

**DOI:** 10.1371/journal.pone.0359838

**Published:** 2026-10-05

**Authors:** Birhanemaskal Malkamu, Abiyu Ayalew, Addisu Melake, Alemie Fentie, Ayenew Assefa, Ayenew Berhan, Birhanu Getie, Birhanu Malede, Biruk Legese, Dagnaw Mersha Tegegne, Dejen Gedamu Damtie, Dessie Tegegne, Eninur Dejen, Fisseha Nigussie Dagnew, Gebrehiwot Berie Mekonnen, Getu Abeje, Habtamu Demlash, Kindie Mitiku, Rahel Mulatie Anteneh, Samuel Berihun Dagnew, Shewaneh Damtie, Tadese Sisay, Teklehaimanot Kiros, Temesgen Lingerh Endalew, Tigabu Eskeziya Zerihun, Mulat Erkihun, Wondimnew Desalegn Addis, Yirgalem Abere, Getaneh Atikilt Yemata, Mahider Shimelis Feyisa

**Affiliations:** 1 Department of Medical Laboratory Sciences, College of Health Sciences, Debre Tabor University, Debre Tabor, Ethiopia; 2 Department of Biomedical Science, College of Health Sciences, Debre Tabor University, Debre Tabor, Ethiopia; 3 Department of Internal Medicine, College of Health Sciences, Debre Tabor University, Debre Tabor, Ethiopia; 4 Department of Social and Public health, College of Health Sciences, Debre Tabor University, Debre Tabor, Ethiopia; 5 Department of Clinical Pharmacy, College of Health Sciences, Debre Tabor University, Debre Tabor, Ethiopia; 6 Department of Pediatric and Child Health Nursing, College of Health Sciences, Debre Tabor University, Debre Tabor, Ethiopia; 7 Department of Epidemiology and Biostatistics, School of Public Health, College of Medicine and Health Sciences, Bahir Dar University, Bahir Dar, Ethiopia; 8 Department of Pediatrics, College of Health Sciences, Debre Tabor University, Debre Tabor, Ethiopia; 9 Department of Adult health Nursing, College of Health Sciences, Debre Tabor University, Debre Tabor, Ethiopia; 10 School of Medical Laboratory Sciences, Asrat Woldeyes Health Sciences Campus, Debre Berhan University, Debre Berhan, Ethiopia; Hawassa University College of Medicine and Health Sciences, ETHIOPIA

## Abstract

**Background:**

University students are vital for Human Papilloma Virus (HPV) prevention, yet comparative data between academic disciplines remain limited. This study compared HPV knowledge, attitudes, and practices (KAP) among female health and Social Science students at Debre Tabor University.

**Objective:**

To compare HPV knowledge, attitudes, and prevention practices (KAP) among female Health and Social Science students and identify sociodemographic predictors of these outcomes.

**Methods:**

**Design:** Institutional-Based, comparative cross-sectional study. **Setting**: Debre Tabor University, Northwest Ethiopia, conducted from June to July 2025.

**Main outcome measures:**

HPV knowledge (good vs. poor), attitudes (good vs. poor), and vaccination practices (good vs. poor). Multivariable logistic regression identified independent predictors of poor outcomes.

**Results:**

Health science students (HSS) reported higher awareness (83.6% vs. 56.7%) and more positive attitudes (57.3% vs. 37.6%) than Social Science students (SSS). However, both groups exhibited poor knowledge: 61.2% of HSS and 80.9% of SSS scored <50% on knowledge questions, and critically low vaccination rates (HSS: 18.2%; SSS: 15.2%). For HSS, lack of prior awareness was the strongest predictor of poor practice (AOR = 7.61, 95% CI = 1.65–35.12). For SSS, second-year students had 3.39 times higher odds of poor practice compared to seniors (AOR = 3.39; 95% CI: 1.04–10.99). SSS predominantly relied on social media (45.0%), while HSS used academic sources (67.5%).

**Conclusion:**

A profound knowledge-practice gap exists regardless of the field of study. High reliance on social media among SSS and low practice among mid-level students suggest that university-based interventions could potentially contribute to national efforts, though achieving population-level targets requires multi-sectoral strategies..

## Introduction

Human Papilloma virus (HPV) is a highly Prevalent viral infection that poses a significant public health threat worldwide, primarily due to its strong association with cervical cancer and other anogenital and oropharyngeal cancers. Globally, cervical cancer remains a major cause of morbidity and mortality among women, particularly in developing countries where the burden of disease is highest [1,2]. In Ethiopia, while national immunization programs have expanded, the burden of cervical cancer remains high, necessitating a deeper look at the ‘last mile’ of prevention-the transition from awareness to clinical practice among highly educated cohorts. The World Health Organization (WHO) has identified HPV as the leading cause of this preventable cancer, and despite the availability of safe and effective HPV vaccines, the global burden remains substantial, highlighting the need for improved prevention strategies [3,4].

Prevention of HPV- related diseases relies on a multifaceted approach that includes comprehensive health education, vaccination, and regular screening. Research has consistently shown that a high level of knowledge and a positive attitude toward HPV and its vaccination are critical precursors to vaccination uptake [5,6]. However, significant disparities exist in awareness and prevention practices, often influenced by socio-demographic factors such as age, residence, marital status, and educational background [7,8]. The academic setting, particularly among young women, is a crucial environment as it offers a unique opportunity to provide directed education and promote preventative behaviors [9]. This study was guided by a comparative empirical framework, focusing on measurable differences in KAP between academic disciplines without imposing a specific behavioral theory. In Ethiopia, a developing country with a high burden of cervical cancer, the implementation of HPV prevention programs faces unique challenges. Recent regional studies have documented general HPV awareness among Ethiopian students [10–12]; however, these findings often treat the student population as a monolithic group. This oversight is significant because it fails to account for the divergent information sources and professional expectations between students in health-related versus non-health-related disciplines. Understanding these factors is essential for designing effective, evidence-based public health interventions.

This study aimed to address this critical evidence gap by providing a, comparative analysis of HPV knowledge, attitudes, and practices among female students at Debre Tabor University. By specifically contrasting Health Science Students (HSS)-who have curriculum-based exposure-with Social Science Students (SSS), who rely predominantly on informal information sources. We hypothesized that Health Science students would demonstrate higher knowledge and more favorable attitudes due to curriculum‑based exposure, but that vaccination uptake would remain low in both groups due to structural barriers. This research identifies the specific points where knowledge fails to translate into practice. Our findings provide the evidence-based rationale needed to transition from generalized public health messaging to discipline-specific institutional strategies, providing baseline data that may inform institutional components of broader national strategies

## Materials and methods

### Study design, area, and period

This institutional-based, comparative cross-sectional study was conducted at Debre Tabor University (DTU) from June to July 2025. DTU is located in Debre Tabor, the capital city of the South Gondar zone in Amhara regional state, approximately 667 kilometers southwest of Addis Ababa, Ethiopia. The university, established in 2008, comprises five colleges, one faculty, and two schools. The study specifically targeted students from two of these colleges: the College of Health Sciences and the College of Social Sciences and Humanities. This setting was selected to allow for a direct comparison of HPV knowledge, attitudes, and practices between students with different academic backgrounds.

This study is reported in accordance with the Strengthening the Reporting of Observational Studies in Epidemiology (STROBE) statement.

Patient and public involvement (PPI) Patients and/or the public were not involved in the design, conduct, reporting, or dissemination plans of this research.

### Study population and sampling

The source population for this study included all female students registered at Debre Tabor University in 2025, while the study population specifically consisted of eligible female students from the College of Health Sciences (HSS) and the College of Social Sciences and Humanities (SSS). A non-probability quota-based convenience sampling approach was employed due to logistical constraints, including the absence of a complete, real-time sampling frame and the restricted data collection window from June to July 2025. Initially, the two colleges were purposively selected to enable a comparative analysis between health-focused and non-health-focused curricula. The required sample size was determined using a single population proportion formula: n = Zα/2^2^ p*(1-p)/d^2^.

A 95% confidence level (Za/2 = 1.96), a 5% margin of error (d = 0.05), and a proportion (p) of 72.7%, representing the prevalence of adequate HPV awareness in a similar university population from a previous study at the University of Gondar and Bahr Dar University, were used [13]. The initial calculated sample size was 305. To account for a 10% non-response rate, an additional 30 students were included, bringing the total sample size to 335 female health science students and 335 Social Science students, for a total of 670 participants. A 100% response rate was achieved as all distributed questionnaires were completed and returned during the supervised data collection sessions

### Data collection

A structured, self-administered questionnaire was used to collect data. The tool was adapted from similar studies and modified after an extensive literature review [13,14]. The questionnaire was designed to capture attitude, knowledge, and practice framed as measuring perceived susceptibility, perceived benefit and actual practice. Its content validity was ensured through an expert review process involving faculty members from the College of Health Sciences. The questionnaire was initially prepared in English, translated into the local language (Amharic), and then back-translated into English by a different translator to ensure consistency.

The questionnaire consisted of three main sections:

1. Socio-demographic data: Age, marital status, religion, residence, academic year, and field of study.
2. HPV Awareness: Questions to assess if students had heard about HPV and their sources of information.
3. Knowledge, Attitudes, and Practices (KAP): Specific questions to measure participants’ knowledge, attitudes, and prevention practices regarding HPV and its vaccination.

A total of 670 eligible female students (335 from the College of Health Sciences and 335 from the College of Social Sciences and Humanities) were included in the study, representing 100% of the calculated target sample size. To ensure the study reached its planned statistical power, recruitment and data collection continued within each academic cohort until the required quota of 335 completed and valid questionnaires per group was achieved.

### Operational definitions

Awareness: In this study, awareness is defined as having previously heard of HPV (measured as a binary ‘Yes/No’ response).

Knowledge: Participants were categorized as having ‘good knowledge’ if they correctly answered greater than or equal to 50% of the knowledge-related questions. Scores less than 50% were categorized as ‘poor knowledge’. The 50% cutoff was chosen based on modified Bloom’s criteria, which categorizes scores below 50% as poor, and is consistent with prior Ethiopian HPV studies, such as Mekonnen et al 2024 [13]

Attitude: Participants’ attitudes were measured using a composite score. Scores were classified as poor attitude for less than 50%, and good attitude equal to or more than 50% of the total attitude score.

Practice: Good practice was defined as having received at least one dose of the HPV vaccine. Participants who had received no doses at all were categorized as having poor practice.“

### Data quality control

To ensure data quality, the questionnaire was pre-tested on 10% of the sample (67) in a different population of engineering students, a population with similar demographics but a different academic background, to ensure clarity and reliability before the main data collection began. The pre-test data were not included in the final analysis. Based on this pre-test, necessary adjustments were made to the tool to improve clarity and validity.

To assess the internal consistency of the questionnaire, Cronbach’s alpha coefficients were calculated for the knowledge and attitude domains separately for Health Science and Social Science student cohorts. The knowledge domain (14 items) demonstrated excellent internal consistency in both groups (Health Science: α = 0.930; Social Science: α = 0.930). The attitude domain (5 items) showed acceptable reliability in both groups (Health Science: α = 0.762; Social Science: α = 0.762). Corrected item-total correlations for all items exceeded 0.30, confirming that each item contributed meaningfully to its respective scale. These results indicate that the questionnaire reliably measured the intended constructs across both student populations.

During the main data collection phase, rigorous quality control was maintained through two principal measures. To assess the consistency of data abstraction and recording, inter-rater reliability (IRR) checks were conducted on the data abstraction process, by two separate, trained data collectors on 5% of the collected questionnaires. The agreement was measured using the Kappa statistic, which yielded a result of kappa = 0.65, indicating substantial agreement. Data Entry Accuracy: All collected data were subjected to double data entry by two independent data clerks. The entries were subsequently compared using validation procedures in SPSS to identify and correct any inconsistencies, ensured an error rate of less than 1%. Furthermore, the principal investigator reviewed all completed questionnaires daily for completeness and consistency. Study participants were clearly informed about the study’s purpose to foster honest responses.

In addition, STROBE (Strengthening the Reporting of Observational Studies in Epidemiology) guidelines were followed throughout the study.

### Ethical approval and consent to participate

Ethical approval and clearance for the study were obtained from the Research and Community Service Corporate Directorate Director, College of Health Science, Debre Tabor University, protocol number 017/2024, at July 20, 2024. The study was conducted in accordance with the Declaration of Helsinki. Informed consent was secured from each respondent after a thorough explanation of the study’s purpose and their rights, including the option to withdraw at any time. All data obtained were kept confidential and private, with no personal identifiers recorded. The privacy and rights of the participants were respected throughout the study.

### Data processing and analysis

All collected data were checked for completeness, coded, and entered into SPSS statistical software version 26.0. Descriptive statistics were used to summarize socio-demographic characteristics, awareness, and KAP levels. For inferential analysis, both bi-variable and multivariable logistic regression models were used to identify factors associated with poor HPV knowledge, attitude, and practice. Variables with a < 0.25 in the bivariable analysis were considered candidates for the multivariable logistic regression model to control for potential confounders. A backward stepwise elimination method was used to identify independent predictors of knowledge, attitude, and practice.

Multicollinearity was assessed using the Variance Inflation Factor (VIF), and the results confirmed the absence of significant collinearity (Max VIF = 1.836). The goodness-of-fit of the multivariable models was evaluated using the Hosmer–Lemeshow test, with a p-value = 0.34 ( > 0.05) indicating an adequate fit. In

The final multivariable model, an Adjusted Odds Ratio (AOR) with a 95% Confidence Interval (CI) was used to measure the strength of association, and a p-value <0.05 was considered statistically significant.

## Results

### Comparative descriptive results

#### Socio-demographic characteristics.

A total of 670 female students participated, with equal representation from Health Science (n = 335) and Social Science (n = 335) streams. The majority of participants in both groups were aged 20–23 years (81.5% and 74.3% respectively) and predominantly from rural residences (56.9% total). (Table 1).

**Table 1:** Socio-demographic characteristics of Female Health Science and Social Science Students at Debre Tabor University.

Variable	Category	Health Science (n = 335)	Social Science (n = 335)	Total (N = 670)
**Age**	20–23	273 (81.5%)	249 (74.3%)	522 (77.9%)
	24–26	62 (18.5%)	86 (25.7%)	148 (22.1%)
**Religion**	Orthodox	286 (85.4%)	248 (74.0%)	534 (79.7%)
	Protestant	39 (11.6%)	44 (13.1%)	83 (12.4%)
	Muslim	10 (3.0%)	18 (5.4%)	28 (4.2%)
	Catholic	0 (0.0%)	25 (7.5%)	25 (3.7%)
**Residence**	Urban	151 (45.1%)	138 (41.2%)	289 (43.1%)
	Rural	184 (54.9%)	197 (58.9%)	381 (56.9%)
**Marital Status**	Single	322 (96.1%)	304 (90.7%)	626 (93.4%)
	Married	9 (2.7%)	26 (7.8%)	35 (5.2%)
	Divorced	4 (1.2%)	5 (1.5%)	9 (1.3%)

### HPV awareness and sources of information among Female Health Science and Social Science Students

A total of 670 participants were surveyed regarding their awareness of HPV. The majority of students (n = 280, 83.6%) among Health Science Students, reported that they had heard of HPV, while a minority (n = 55, 16.4%) had not (Table 2). Among Social Science Students, social media (n = 86, 45%) is the highest source of information.

**Table 2:** HPV awareness and sources of information among Female Health Science and Social Science Students (N = 670).

Variable	Category	Health Science (n = 335)	Social Science (n = 335)
**Heard about HPV**	Yes	280 (83.6%)	190 (56.7%)
	No	55 (16.4%)	145 (43.3%)
Source of information*	Academic	189 (67.5%)	27 (14.2%)
	Health center	48 (17.1%)	38 (20.0%)
	Social media	33 (11.8%)	85 (44.7%)
	Friends	6(2.1%)	27 (14.2%)
	Family	4 (1.4%)	13 (6.8%)

Note: The percentages for ‘Source of Information’ are calculated based on the subset of participants who reported prior awareness of HPV (n = 280 for Health Science and n = 190 for Social Science). This explains the variation in denominators compared to the total sample size (N = 670).

### HPV knowledge, attitude, and practice level of Female Health Science and Social Science Students

The overall knowledge, Attitude, and Practice level regarding HPV among the participating Female Health Science and Social Science Students was assessed (Table 3).

**Table 3:** Distribution of HPV knowledge, attitude, and vaccination practice level among Female Health Science Students at Debre Tabor University.

Variable	Category	Health Science (n = 335)	Social Science (n = 335)
**Knowledge level**	Good Knowledge	130 (38.8%)	64 (19.1%)
	Poor Knowledge	205 (61.2%)	271 (80.9%)
**Attitude level**	Good Attitude	192 (57.3%)	126 (37.6%)
	Poor Attitude	143 (42.7%)	209 (62.4%)
**Vaccination Practice level**	Good Practice	61 (18.2%)	51 (15.2%)
	Poor Practice	274 (81.8%)	284 (84.8%)

Pearson’s chi-square tests were performed to formally compare Health Science and Social Science students across key outcomes. Health Science students had significantly higher HPV knowledge (38.8% vs. 19.1% good knowledge; χ² = 31.61, df = 1, p < 0.001) and more favorable attitudes towards HPV vaccination (57.3% vs. 37.6% good attitude; χ² = 26.07, df = 1, p < 0.001) compared to Social Science students. However, vaccination practice did not differ significantly between the two groups (18.2% vs. 15.2% vaccinated; χ² = 1.07, df = 1, p = 0.300).

### Factors associated with HPV knowledge, attitude and practice of Female Health Science Students

To identify factors associated with HPV knowledge, attitude, and practice, logistic regression analyses were performed exclusively on the data collected from the Health Science student group (N = 335). This approach was taken to investigate the determinants of these outcomes with a population directly relevant to health-related interventions and education

### Factors associated with HPV knowledge and practice of Female Health Science Students

Multivariable logistic regression was performed to identify independent predictors of poor HPV knowledge and practice among Health Science students. After adjusting for potential confounders, including age, residence, and academic department, prior awareness of HPV emerged as the most significant determinant for both knowledge and behavior (Table 4).

**Table 4:** Multivariable logistic regression of factors associated with poor HPV knowledge and poor practice among Female Health Science Students (n = 335).

Variable	Category	Poor Knowledge AOR (95% CI)	p-value	Poor Practice AOR (95% CI)	p-value
**Heard about HPV**	No	6.96 (2.29–21.15)	0.001*	7.61 (1.65–35.12)	0.01*
	Yes (Ref)	1		1	
**Age**	20–23	0.55 (0.27–1.10)	0.09	0.94 (0.40–2.20)	0.89
	24–26 (Ref)	1		1	
**Residence**	Urban	1.35 (0.82–2.23)	0.23	0.87 (0.47–1.59)	0.64
	Rural (Ref)	1		1	
**Academic Year**	2nd year	3.21 (0.99–10.32)	0.05*	1.52 (0.41–5.62)	0.53
	3rd year	0.83 (0.28–2.45)	0.74	2.61 (0.74–9.20)	0.13
	4th year	2.28 (0.79–6.57)	0.13	1.42 (0.45–4.53)	0.55
	5th year (Ref)	1		1	
**Department**	Anesthesia	0.77 (0.20–2.97)	0.70	0.48 (0.08–2.95)	0.43
	Medical Lab	0.77 (0.21–2.83)	0.69	1.01 (0.14–6.91)	0.98
	Pharmacy	0.95 (0.30–3.02)	0.93	0.72 (0.13–3.86)	0.70
	Comp. Nursing	0.86 (0.24–3.05)	0.81	0.22 (0.04–1.20)	0.08
	Ped. Nursing	0.82 (0.21–3.21)	0.78	0.64 (0.09–4.47)	0.65
	Medicine	0.63 (0.20–1.98)	0.43	0.50 (0.10–2.51)	0.40
	Midwifery	0.66 (0.20–2.13)	0.48	1.32 (0.22–7.79)	0.76
	Psych. Nursing (Ref)	1		1	

* Denotes statistical significance at *P* < 0.05.

It is worth noting that certain predictors, particularly ‘prior awareness of HPV’ (Heard about HPV), demonstrated wide 95% confidence intervals (AOR = 7.61, 95% CI: 1.65–35.12). This may indicate a lower level of precision in the point estimate for this variable, likely due to small sample sizes within specific subgroups; therefore, these associations should be interpreted with caution.

The multivariable logistic regression models used to identify predictors of HPV knowledge and practice among Health Science students were assessed for statistical validity. The model for HPV knowledge demonstrated a good fit to the data (Hosmer–Lemeshow test: X^2^ = 24.52, p = 0.336), while the model for prevention practice also showed an adequate fit (Hosmer–Lemeshow test: X^2^ = 14.66, *P* = 0.066). Furthermore, no significant multicollinearity was detected among the independent variables, with a maximum Variance Inflation Factor (VIF) of 1.836. These diagnostics confirm that the models are reliable for identifying the independent factors associated with these outcomes.

### Associated factors with HPV attitude of Female Health Science Students

As illustrated in Table 5, the bivariable logistic regression analysis revealed that none of the investigated socio-demographic factors or prior awareness levels were significantly associated with the attitude of Health Science students toward HPV prevention (*P* > 0.05). Regarding students’ academic year: When using the second-year students as a reference group, the bivariable logistic regression showed that higher academic years tended to have higher odds of a good attitude, particularly among fifth-year students (COR = 2.46, 95 CI: 0.97–6.23); however, this association did not reach statistical significance (*P* = 0.06).

**Table 5:** Binary logistic regression outcome of HPV Attitude of Female Health Science Students.

Variable	Category	Level of Attitude		COR (95% CI)	p value
		Good attitude	Poor attitude		
Heard about HPV	Yes	36	19	0.66 (0.36-1.21)	0.18
	No (Ref)	156	124	1	
Age	20-23	160	113	0.75 (0.43-1.31)	0.32
	24-26(Ref)	32	30	1	
Residence	Urban	84	67	1.13 (0.73-1.75)	0.57
	Rural (Ref)	108	76	1	
Academic Year	2^nd^ Year	75	44	1	–
	3^rd^ Year	55	53	1.64 (0.97-2.79)	0.07
	4^th^ Year	53	33	1.06 (0.60-1.88)	0.84
	5^th^ Year (Ref)	9	13	2.46 (0.97-6.23)	0.06
Department	Anesthesia	12	12	0.91 (0.28-2.93)	0.87
	Medical laboratory	20	12	0.54 (0.18-1.66)	0.29
	Pharmacy	35	21	0.54 (0.19-1.50)	0.24
	Comprehensive nursing	24	16	0.61 (0.21-1.76)	0.36
	Pediatric nursing	17	9	0.48 (0.15-1.56)	0.22
	Medicine	40	43	0.97 (0.37-2.55)	0.96
	Midwifery	34	19	0.51 (0.18-1.41)	0.19
	Psychiatric nursing	10	11	1	

P < 0.05, Ref- reference.

Note: For multivariable logistic regression, the reference category for academic year was set to 2^nd^ year to provide a more intuitive interpretation of changes in attitudes and practices as students’ progress through their academic program.

### Factors associated with HPV knowledge, attitude and practice of Female Social Science Students

#### Factors associated with HPV knowledge of Female Social Science Students.

the multicollinearity assessment showed high tolerance for all predictors, with VIF values ranging from 1.013 to 1.252, indicating the independence of the variables included in the final model.

The multivariable model for knowledge of Social Science students showed a significant Hosmer–Lemeshow result (X^2^ = 26.26, *P* = 0.001). However, the model demonstrated an overall classification accuracy of 80.3% and a Nagelkerke R square of 0.237, suggesting that the model explains 23.7% of the variance in knowledge and remains a useful tool for identifying key predictors. Furthermore, no evidence of multicollinearity (Max VIF = 1.25).

The multivariable model for prevention practice among Social Science students demonstrated a robust fit to the data (Hosmer–Lemeshow test: X^2^ = 4.10, *P* = 0.77). Multicollinearity assessment showed high tolerance for all predictors, with VIF values ranging from 1.01 to 1.21, indicating the independence of the variables.

In the final model for practice, residence was identified as the only significant predictor. Students residing in urban areas had significantly lower odds of poor prevention practice compared to their rural counterparts (AOR = 0.54, 95% CI: 0.29–0.99, *P* = 0.047 round to 0.05). Other factors, including academic year and age, did not show statistically significant associations with prevention practice in this cohort (p > 0.05) (Table 6).

**Table 6:** Multivariable logistic regression of factors associated with poor HPV knowledge and poor practice among Female Social Science Students (n = 335).

Variable	Category	Poor Knowledge AOR (95% CI)	p-value	Poor Practice AOR (95% CI)	p-value
**Heard about HPV**	Yes	1.74 (0.91–3.34)	0.10	0.90 (0.48–1.68)	**0.73**
	No (Ref)	1		1	
**Age**	20–23	0.25 (0.07–0.93)	0.04*	1.01 (0.49–2.10)	0.97
	24–26 (Ref)	1		1	
**Residence**	Urban	1.27 (0.66–2.48)	0.47	0.54 (0.29–0.99)	0.05*
	Rural (Ref)	1		1	
**Academic Year**	1st year	0.35 (0.08–1.43)	0.14	2.88 (0.35–23.63)	0.32
	2nd year	0.12 (0.05–0.31)	<0.001*	1.50 (0.64–3.50)	0.35
	3rd year	1.48 (0.49–4.39)	0.48	1.44 (0.68–3.06)	0.34
	4th year (Ref)	1		1	
**Department**	Management	1.29 (0.31–5.32)	0.72	—	—
	Economics	2.84 (0.70–11.51)	0.14	—	—
	Accounting	0.92 (0.23–3.57)	0.91	—	—
	Tourism	3.79 (0.75–19.12)	0.11	—	—
	English/Lit	2.03 (0.43–9.65)	0.37	—	—
	History	0.78 (0.19–3.12)	0.72	—	—
	Sociology	0.98 (0.24–3.91)	0.97	—	—
	Psychology (Ref)	1		—	—

* The Exact **P**- Value of Urban residence is 0.047 round to 0.05.

(—) indicates variables that were not entered into the multivariable model because they did not meet the significance threshold (p < 0.25) during bivariable analysis.

### Associated factors with HPV attitude of Female Social Science Students

A bi- variable logistic regression analysis was conducted to identify factors associated with poor attitude towards HPV among Social Science students of Debre Tabor University. None of the variables, including age, residence, marital status, academic year, department, and having heard about HPV, showed a statistically significant association with poor attitude (Table 7).

**Table 7:** Bi-variable Logistic Regression Analysis of Factors Associated with Attitude towards HPV among Social Science Students at Debre Tabor University, Debre Tabor, Ethiopia, July 2025.

Variable	Category	Level of Attitude		OR (95% CI)	P value
		Good	Poor		
Heard about HPV	Yes	70	120	1.08 (0.69-1.68)	0.74
	No	56	89		1
Age	20-23	90	159	1.27 (0.77-2.10)	0.35
	24-26	36	50		
Residence	Urban	51	87	1.05 (0.66-1.64)	0.84
	Rural	75	122		1
Academic Year	2^nd^ year	6	7	0.62 (0.20-1.94)	0.47
	3^rd^ year	28	60	1.13 (0.64-2.00)	0.67
	4^th^ year	45	53	0.62 (0.36-1.06)	0.08

*P < 0.05, Ref- reference.

## Discussion

Our study revealed a significant disparity in basic HPV awareness: 83.6% of Health Science students (HSS) had heard of HPV, compared to only 56.7% of Social Science Students (SSS). These findings contrast with the study by Yohannes et al. (2023) among adolescent school girls in Ambo Town, which reported an awareness level of 47.3%. The higher awareness in our cohorts, particularly among Health Science Students, likely reflects the cumulative effect of academic exposure and age. However, the relatively lower awareness among our Social Science Students (56.7%) suggests that the awareness gains seen in earlier school years, as documented by Yohannes et al., are not being sufficiently sustained or expanded for Social Science students upon entering higher education [11].

However, despite this difference in awareness, the significant knowledge- practice gap in our study reveals a key challenge for HPV prevention. Consistent with prior research in developing nations, a substantial portion of our study population demonstrated a poor level of HPV knowledge [14,15]. This deficit was particularly pronounced among Social Science students, where over 80% were categorized as having poor knowledge. While health science students exhibited a higher proportion of good knowledge, a majority (61.2%) still fell into the poor knowledge category. This finding is significant as it challenges the assumption that students in health-related fields are inherently well-informed on public health issues like HPV.

Significant disparities in primary information sources likely drive the observed knowledge gap, with health science students (HSS) primarily utilizing academic curriculum (67.5%) while Social Science students (SSS) predominantly rely on social media (45.0%). Despite these differences, a critical knowledge-practice gap persists across both cohorts; for example, 57.3% of HSS demonstrated positive attitudes toward HPV, yet vaccination remains exceptionally low at 18.2%. Similarly, SSS students exhibited low positive attitudes (37.6%) and comparably low practice (15.2%), suggesting that substantial non-cognitive barriers-such as cost, access, and logistical hurdles-prevent favorable sentiments from translating into preventive action. [16–18].

Furthermore, while Yohannes et al. (2023) identified that positive attitudes toward vaccination were high among younger adolescents (72.8%), our study found a significant decay in these sentiments among Social Science University Students (37.6%) [11].This cross- sectional finding suggests that positivity may differ by academic year, but longitudinal data are needed to confirm a true decline.. This highlights that university-based interventions cannot assume that awareness built during primary or secondary schooling is sufficient to drive vaccination practice in young adulthood.

Our comparative analysis, identified distinct predictors of HPV knowledge and prevention practice for each student group, emphasizing the need for educational strategies. Our finding that HPV knowledge is low among university students aligns with similar studies conducted at Addis Ababa University and Jimma University [19,20]

Predictors for poor KAP varied significantly by discipline; for health science students (HSS), a lack of prior HPV awareness was the strongest determinant of both poor knowledge (AOR = 6.96, 95% CI: 1.65–35.12: p < 0.001) and poor practice (AOR = 7.61, 95% CI:1.65–35.12: p = 0.01), confirming that basic awareness is foundational yet currently insufficient in depth. Conversely, Social Science students (SSS) displayed a paradoxical trend where being in the 2nd academic year (AOR = 0.12,95%CI: 0.05–0.31: p < 0.001) and the 20–23 age group (AOR = 0.25,95% CI: 0.07–0.93: p = 0.04) were protective against poor knowledge compared to seniors. This suggests that while initial, potentially social-media-driven exposure provides early awareness, this knowledge is not reinforced as students’ progress through a non-health-related curriculum, creating an observed gap in later academic years.

Unlike Getaneh et al. (2021), who identified that being in a senior year of study was a positive predictor for screening knowledge [12], our comparative analysis found a more nuanced trend. In our Social Science cohort, seniority did not significantly improve vaccination practice, whereas it did for Health Science students. This may indicate that the ‘academic maturation’ effect noted in the Gondar study may be limited to students in health-related fields. For students in the Social Sciences, the observed gap in later academic years persists throughout their university tenure, highlighting a missed opportunity for institutional health promotion that the Getaneh study did not explore.

Among Social Science students (SSS), residence was the sole significant predictor of HPV prevention practices. Students from urban areas were significantly less likely to report poor practice (AOR = 0.54, 95% CI: 0.29–0.99, *P* = 0.047) compared to their rural counterparts. This suggests that urban residence may afford better access to information or vaccination services, whereas rural students face geographic or structural barriers to care.

While academic year was a significant predictor of knowledge, it did not statistically influence practice in this cohort (*P* > 0.05). This “knowledge-practice gap” emphasizes that increasing academic awareness alone is insufficient for non-health tracks; targeted interventions must address the practical barriers faced by rural students. Furthermore, while this study focused on academic]discipline, the Ethiopian context necessitates exploring unmeasured cultural and religious influences on sexual health. Expanding future research to include male students remains critical for achieving community-wide immunity and meeting the WHO “90-70-90” targets [21].

While Yohannes et al. (2023) noted that teachers and health workers were primary information sources for younger girls, our results show a shift in the information landscape for university students. For our Social Science cohort, social media (45.0%) has overtaken institutional sources. This evolution in information-seeking behavior between adolescence and university age is a critical finding of our study, identifying social media reliance was associated with lower knowledge scores; this finding generates the hypothesis that social media content may lack sufficient clinical detail. We did not directly assess misinformation exposure; this interpretation is speculative and requires confirmation.

Our findings regarding the disparity in attitudes between HSS (55.4% positive) and SSS (37.6% positive) build upon the regional evidence provided by Getaneh et al. (2021). In their study at the nearby University of Gondar, they reported that only 13.7% of students had ever undergone cervical cancer screening, despite a majority having heard of the disease. While Getaneh et al. highlighted a massive gap in screening utilization among a general undergraduate population, our study identifies that a similar, discipline-specific gap exists for primary prevention (vaccination). Specifically, we demonstrate that even when students are in an environment with high screening awareness (like Northwest Ethiopia), those in Social Science tracks remain significantly more hesitant toward vaccination than their health science peers. This suggests that regional awareness of screening does not ‘spill over’ into vaccine acceptance without discipline-specific curriculum intervention.

Our finding of 18.2% vaccination uptake is strikingly similar to a 2022 study in Bahir Dar City, which reported a 16% uptake among female preparatory students (Likneh et al., 2022) [22]. This suggests that as female students transition from primary education to higher levels in the Amhara region, vaccination coverage remains consistently low. The significantly higher uptake (66.5%) reported in North Shoa (Kassa et al., 2021) was observed among primary school girls, who are the primary target of Ethiopia’s school-based National Expanded Program on Immunization [23]. In contrast, our study population consists of university students who likely missed these initial school-based campaigns and face greater barriers to accessing the vaccine as young adults.

Our findings of low vaccination rates among university students mirror trends observed across other Low- and Middle-Income Countries (LMICs). For instance, studies in Nigeria have noted uptake rates ranging from 1.5% to 20.4% among university students, with vaccine unavailability (66.7%) and lack of awareness (63.2%) identified as primary barriers [24]. In India, a study among female nursing and paramedical students reported only 7.7% vaccination coverage, with high cost (64.9%) and lack of information (25.4%) as major obstacles [25]. Similarly, in Malaysia, 40% of university students had not received the vaccine and had no scheduled appointment, highlighting that university students often fall into a ‘catch-up’ gap where the absence of campus-based vaccination clinics hinders preventive action [26]. This highlights a universal challenge: the transition to higher education often results in a loss of momentum for series completion or initial uptake if not supported by institutional health services.

Ethiopian national data from 2025 reports a 30.8% HPV vaccination uptake among school girls, while a meta-analysis estimated regional acceptance at 41.46% (PMA Ethiopia, 2025; BMC Public Health, 2023) [27,28]. Our lower reported practice (15.2%–18.2%) suggests that actual uptake in the university setting is lagging behind national expectations, indicating a critical need for university-based catch-up programs.

The low vaccination uptake, despite positive attitudes, highlights a critical challenge for achieving the WHO’s Global Strategy to Accelerate the Elimination of cervical cancer [29]. This global strategy aims to achieve a “90-70-90” target by 2030, which includes vaccinating 90% of girls by age 15 [30,31]. While Ethiopia has demonstrated a strong commitment to this goal by incorporating the HPV vaccine into its national immunization program, recent coverage rates remain far below this target, with administrative coverage for the two-dose schedule at 74% in 2022 and with the country having since shifted to a single dose schedule [32,33]. The low vaccination rate in our study is not merely a result of poor individual knowledge, but a reflection of systemic policy barriers. Ethiopia’s HPV vaccine rollout primarily targets 9–14-year-olds; university students are not currently a priority group for routine national campaigns, resulting in low coverage in this age cohort. Therefore, for Ethiopia to approach its ‘90-70-90’ targets, national strategies must be complemented by localized, data-driven interventions at the institutional level.

The discrepancy between the positive attitudes observed in our study and the low vaccination rates is a major finding, mirroring a well-documented challenge in low- and middle-income countries (LMICs) [34,35]. This gap suggests that factors beyond simple awareness are at play. In the Ethiopian context, these barriers include logistical and financial hurdles such as inconsistent vaccine supply, inadequate cold chain infrastructure, and the high cost of vaccination, which can be prohibitive even when the vaccine is available [36]. Vaccine hesitancy, fueled by misinformation and social stigma surrounding sexual health, also remains a significant barrier to uptake in many communities [37,38].

Our findings therefore suggest that university-based interventions could serve as a valuable platform for achieving national and global goals by addressing both the attitudinal and logistical barriers. Implementing targeted educational campaigns in these settings could not only raise awareness but also directly address misinformation and hesitancy. Furthermore, integrating vaccination services directly within the university health system could improve accessibility and overcome logistical challenges, thereby converting positive attitudes into tangible prevention practices. This approach aligns with the National Cancer Control Plan of Ethiopia (2025–2029), which prioritizes increasing access to HPV vaccination as a key strategy for reducing the national cancer burden [39].

### Study limitations and strengths

This study employed a comparative cross-sectional design, which provides a valuable snapshot of the current situation but cannot establish causal relationships between the variables. Additionally, the self-reported nature of our data is also subject to recall and social desirability bias. Furthermore, our operational definition of ‘good practice’ was receiving at least one dose of the HPV vaccine; this was a pragmatic choice, but it does not differentiate between partial and full vaccination according to the national schedule at the time of vaccination. Nevertheless, a key strength of this research is its comparative nature, which provides a detailed, nuanced understanding of the differences in KAP and associated factors between two distinct student populations, a critical step for developing effective, evidence-based interventions.

While this study achieved its target sample size with a 100% response rate, the use of non-probability convenience sampling is a limitation that may impact internal validity. This approach introduces a potential for selection bias, as students who were present in class during the data collection window were the ones recruited, potentially differing from those who were absent. To ensure the target was met, recruitment continued within each department until the pre-specified quota of 335 students per college was achieved. This high level of participation ensures that the captured data represents the majority of students active during the study period, providing a solid foundation for this comparative analysis.

Our study did not measure psychological constructs such as perceived susceptibility or barriers to vaccination, which may mediate the knowledge-practice gap observed. Future research should incorporate validated Health Belief Model instruments. In addition, prevention practices were measured solely by vaccination uptake; screening behaviors (e.g., Pap smears) and vaccination intentions were not assessed. Consequently, we cannot determine whether current attitudes will predict future screening or vaccine uptake.

Furthermore, some of the measures of association in the multivariable models exhibited wide confidence intervals, particularly concerning vaccine practice and awareness. These results suggest a limited degree of statistical precision for certain sub-categories. While the associations remain statistically significant, the instability of these specific estimates should be considered when generalizing these findings to broader populations.

Our cross-sectional design was unable to differentiate between students who were ineligible for vaccination, those who were hesitant, and those who lacked access. A future study using qualitative methods or a more detailed survey could provide critical insights into the specific reasons for low vaccination rates. Future longitudinal studies should incorporate cervical cancer screening behaviors and vaccination intentions to provide a more holistic assessment of HPV prevention strategies among university students.

## Conclusion and implications

Based on our observational findings, we recommend university-wide HPV initiatives at Debre Tabor University to address pervasive knowledge deficits, particularly among Social Science students (80.9% poor knowledge). For health science cohorts, interventions should focus on curriculum-driven strategies to bridge the attitude-practice gap, while Social Science disciplines require tiered, peer-led campaigns and mandatory foundational public health modules to counter lower knowledge levels observed in later academic years. Institutional strategies could complement national efforts, though cross-sectional KAP studies cannot directly measure progress toward elimination targets [40]. Therefore, future research must utilize qualitative methods to investigate the socio-cultural drivers of vaccine hesitancy. Such data-driven, discipline-specific approaches are vital for converting awareness into tangible preventive actions and reducing the national cervical cancer burden in Ethiopia.

## Supporting information

S1 Data De-identified dataset for Health Science students (n = 335). This SPSS file contains the raw data used for the comparative analysis of HPV knowledge, attitudes, and prevention practices, including socio-demographic characteristics, awareness, knowledge scores, attitude scores, and vaccination status.
(SAV)

S2 Data De-identified dataset for Social Science students (n = 335). This SPSS file contains the raw data used for the comparative analysis of HPV knowledge, attitudes, and prevention practices, including socio-demographic characteristics, awareness, knowledge scores, attitude scores, and vaccination status.
(SAV)

## Abbreviations

AOR: Adjusted Odds Ratio
DTU: Debre Tabor University
HBM: Health Belief Model
HPV: Human Papillomavirus
WHO: World Health Organization
OR: Odds Ratio
LMICs: Low- and middle-income countries
SSS: Social Science students
HSS: Health science students
