# Supplementary material for: Knowledge, attitudes, and prevention practices regarding human papillomavirus among health and social science female students in Northwest Ethiopia: A comparative cross-sectional study
Source: PLoS One. 2026 Oct 5;21(10):e0359838. doi: 10.1371/journal.pone.0359838 (PMC13637910; doi:10.1371/journal.pone.0359838)
Supplement: Supplementary file 1 [file pone.0359838.s005.pdf]

1. The reference category for academic year is inconsistent between Table 5 and the accompanying note. The table appears to use fifth year as the reference category, whereas the note states that second year was used as the reference. Please verify the regression coding and revise the table, text, and footnote to ensure complete consistency.

2. Several regression estimates, particularly for prior HPV awareness and academic department, have wide confidence intervals. The authors should report the underlying cell frequencies and assess whether sparse data or separation may have contributed to the instability of the estimates.

3. In table 6, Heard about HPV p value was unnecessarily reported as **bold** format
