# Supplementary material for: Knowledge, attitudes, and prevention practices regarding human papillomavirus among health and social science female students in Northwest Ethiopia: A comparative cross-sectional study
Source: PLoS One. 2026 Oct 5;21(10):e0359838. doi: 10.1371/journal.pone.0359838 (PMC13637910; doi:10.1371/journal.pone.0359838)
Supplement: Supplementary file 2 [file pone.0359838.s004.docx]

Dear Editor and Reviewers, on behalf of the authoring team, I would like to express our sincere gratitude for the constructive feedback provided during the initial review of our manuscript. In this revised version, we have not only addressed each of the reviewers' specific comments but have also undertaken a comprehensive internal audit to enhance the transparency, statistical robustness, and methodological clarity of our findings.

**Major Issues Requiring Revision**

1. Study Design and Conceptual Clarity

The manuscript repeatedly describes the study as a “prospective cross-sectional” design, which is methodologically incorrect. A cross-sectional study is not prospective unless repeated measures or follow-up are involved.

**Response**- the authors agree on the above comment; we removed the term ‘prospective’

Please revise this terminology consistently throughout the manuscript.

The Health Belief Model (HBM) is mentioned but not clearly operationalized in the analysis.

Either incorporate measurable HBM constructs (e.g., perceived susceptibility, barriers) into the analysis or remove/reduce this theoretical framing.

**Response-** We have removed the reference to the Health Belief Model, as our survey did not directly operationalize its constructs. The manuscript now presents the findings without theoretical framing, which we believe improves clarity and honesty regarding our data's limitations

2. Sampling Procedure and Risk of Bias

There are critical inconsistencies and missing details regarding sampling:

Contradiction between multi-stage/random sampling and convenience sampling

Missing essential elements:

Sampling frame

Stage-wise selection procedures

Proportional allocation

Response rate

These gaps raise concerns about selection bias and internal validity.

You must:

Clearly describe the exact sampling procedure step-by-step

Resolve inconsistencies across sections

Explicitly acknowledge limitations if non-probability sampling was used

**Response-** We have revised the Sampling section to accurately reflect the use of convenience sampling due to the lack of a real-time sampling frame, resolving the previous contradiction between random and convenience methods. We have clarified the participant flow in the Data collection section line 161 to 164. We employed a quota-based recruitment strategy where data collection was maintained within each academic cohort until the pre-calculated sample size of 335 per group (Total N=670) was reached. This ensured the study achieved 100% of its intended sample size to maintain statistical power. We have also updated the Limitations section line 444 to 451 to explicitly acknowledge the selection bias inherent in convenience sampling and how it relates to the internal validity of our findings.

3. Outcome Definitions and Measurement

Several conceptual and operational issues were identified:

The classification of knowledge and attitude (e.g., ≥50% cut-off) appears arbitrary

Provide justification or cite validated instruments

The statement that >60% reflects “poor knowledge” requires clarification and justification

The definition of “practice” is inconsistent:

Defined as full vaccination (two doses) in one section

Later described as at least one dose

Measuring practice solely through vaccination is too narrow

Consider including screening behavior or intention if data are available.

**Response**- We have addressed these conceptual concerns as follows:

Standardized Definition: We have standardized the definition of "Practice" as receiving at least one dose of the HPV vaccine. This definition is now used consistently across the Methods (Line 177to 179), Results, and Discussion.

Acknowledging Scope: We agree that including screening and intention data would have strengthened the study. Since this data was not collected, we have explicitly acknowledged this as a limitation (Line 414 to 417).

Future Recommendations: We have added a recommendation (Line 421-423) for future research to include screening behaviors and behavioral intentions to provide a more comprehensive view of HPV prevention.

4. Statistical Analysis and Reporting

The manuscript lacks key epidemiological reporting standards:

No clear explanation of:

Variable selection criteria (e.g., p-value thresholds such as p<0.25)

Model-building strategy (stepwise vs. forced entry)

Missing diagnostics:

Multicollinearity assessment (e.g., VIF, tolerance)

Model fit (e.g., Hosmer–Lemeshow test, pseudo R²)

Additionally:

Some estimates show wide confidence intervals, indicating instability

Interpret cautiously and acknowledge limited precision

**Response**- We have comprehensively revised the statistical reporting to adhere to PLOS ONE’s standards: Variable Selection: We have specified that variables with p < 0.25 in bivariable analysis were entered into the multivariable model using backward stepwise elimination (Methods, Lines 215–226). Diagnostics: We now report Hosmer–Lemeshow test results, Nagelkerke R², and Classification Accuracy for all models. For the SSS knowledge model, we have justified its use despite the H-L result by highlighting its 80.3% predictive accuracy. Multicollinearity: We have confirmed model stability with VIF values for all predictors (HSS Max VIF = 1.836; SSS Max VIF = 1.252). Precision: We have added cautionary statements regarding variables with wide confidence intervals in both the Results and Limitations.

5. Results Presentation

Inconsistencies in reported sample size (e.g., 335 vs. 670)

Unclear denominators (e.g., source of information n=286)

Table issues:

Poor formatting and unclear structure (e.g., Table 5)

Inappropriate reference category (e.g., use of 5th year instead of 1st year)

Incorrect p-value reporting (e.g., “0.00” should be reported as “<0.001”)

Revise all tables for clarity, accuracy, and consistency with journal standards.

**Response**- We thank the editor for the constructive feedback regarding our statistical reporting. We have revised the Data Processing and Analysis section (Lines 215 to 224) and the Results section (Lines 262 to 269 and 288 to 289) to incorporate these key epidemiological standards:

Variable Selection and Model Building: We have clarified that variables with a p-value < 0.25 in the bivariable analysis were selected as candidates for the multivariable logistic regression. A backward stepwise elimination method was employed to identify independent predictors and control for potential confounders.

Multicollinearity Assessment: We performed diagnostic checks for multicollinearity using the Variance Inflation Factor (VIF). All values were well below the threshold of 10 (Health Science VIF range: 1.02–1.84; Social Science VIF range: 1.01–1.25), confirming the independence of the predictors.

Model Fit and Diagnostics: We have now reported the Hosmer–Lemeshow (H-L) test and Nagelkerke R Square for all multivariable models to demonstrate model fitness:

Health Science Cohort: The models showed adequate fit for both Knowledge (p = 0.336) and Practice (p = 0.066).

Social Science Cohort: The Practice model showed an excellent fit (p = 0.858). While the Knowledge model yielded a significant H-L result (p = 0.001), we confirmed its predictive utility through a high overall classification accuracy of 80.3% and a Nagelkerke R Square of 0.237.

Interpretation of Confidence Intervals: We acknowledge that some variables, such as 'Heard about HPV' in Table 4, exhibited wide 95% Confidence Intervals (CI: 1.65–35.12). We have added a cautionary statement in the Results (Lines 262 to 265) and Limitations sections (Lines 449 to 453) acknowledging that these specific estimates have limited precision and should be interpreted with caution.

Refined P-value Reporting: All p-values previously reported as "0.000" have been corrected to < 0.001 throughout the manuscript and tables.

Sample Size Consistency: We have updated the text and Table 2 to consistently reflect the total sample (N=670) and the equal distribution between the two study cohorts (n=335 each).

Denominator Clarification: We have clarified that the denominator for 'Source of Information' is derived only from participants with prior HPV awareness (n=470 total). We have added a specific footnote to Table 2 to explain this skip-logic, confirming that no data was missing, but rather that the question was only applicable to aware participants.

Table 5 Refinement: We have reformatted Table 5 for clarity and removed redundant descriptions from the text, as suggested.

6. Interpretation and Overstatement of Findings

The manuscript makes causal and temporal interpretations (e.g., knowledge decay, “window of disengagement”) that are not supported by cross-sectional data

Claims regarding contribution to Ethiopia’s 90–70–90 targets should be substantially toned down

Statements about misinformation and behavioral trends are speculative and should be clearly framed as such or removed

**Response**- We have systematically toned down all causal and overreaching claims throughout the manuscript. First, we replaced causal language such as "knowledge decay" and "window of disengagement" with descriptive phrasing (e.g., "lower knowledge levels observed in later academic years") and added a disclaimer that these are hypotheses requiring longitudinal testing, as cross-sectional data cannot establish temporal trends. Second, we softened claims regarding Ethiopia's 90-70-90 targets: "requires" was changed to "could potentially contribute to," and we explicitly state that cross-sectional KAP studies cannot directly measure progress toward elimination targets. Third, we reframed speculation about misinformation as hypotheses (e.g., "may be associated with," "generates the hypothesis that") and added an explicit acknowledgment that we did not directly measure misinformation exposure. Finally, we added a new sentence to the Limitations section stating that all observed associations are correlational and causal inferences cannot be drawn. All changes are highlighted in the revised manuscript.

7. Discussion – Depth and Context The discussion requires strengthening:

Expand literature comparison, especially: Studies from Africa and other LMICs University-based HPV interventions

Provide contextual discussion of: HPV vaccine availability in Ethiopia

National rollout status

Cost and policy barriers

Current vaccination rates (18.2% and 15.2%) are very low but insufficiently contextualized

**Response**- We have revised the Discussion (Lines 406 to 418 and 426 to 429) to provide a rigorous contextualization of our vaccination rates using recent Ethiopian literature:

Regional Alignment: We noted that our results align with findings from Bahir Dar (16%), confirming the validity of our data in the Amhara region.

Target Group Disparity: We contrast our 18.2% rate with the 66.5% reported in primary schools (Kassa et al., 2021), explaining that university students fall into a "policy gap" as they are outside the primary target age for national school-based campaigns.

National Comparison: We have benchmarked our findings against the 2025 PMA national survey (30.8%) and the 2023 regional meta-analysis (41.46%), highlighting that university-aged women represent a underserved population in Ethiopia’s current vaccine rollout strategy.

8. Data Availability (PLOS ONE Policy Compliance)

The statement “data available upon reasonable request” does not comply with PLOS ONE policy

You must:

Deposit data in a public repository, OR

Provide a clear and justified restriction with an access mechanism

**Response-** Thank you for this guidance regarding the PLOS ONE Data Availability Policy. We have updated our statement and our submission to ensure full compliance. We have now provided all relevant raw data as Supporting Information files.

These files include the fully de-identified datasets for both the Health Science and Social Science student cohorts, allowing for complete replication of our reported results, including the multivariable logistic regression and model diagnostics (VIF and Hosmer-Lemeshow tests).

9. Thank you for stating the following in your Ethics Statement that “Ethical approval and clearance for the study were obtained from the Research and Community Service Corporate Directorate Director, College of Health Science, Debre Tabor University, protocol number 014/2025, at May 15, 2025."

During our initial internal evaluation of the manuscript, we noticed that in your manuscript you reported "Ethical approval and clearance for the study were obtained from the Research and Community Service Corporate Directorate Director, College of Health Science, Debre Tabor University, protocol number 017/2024, at July 20, 2024. "

Please clarify the approval number that was provided for this study and provide the correct approval from your ethics committee for this study in the ethics statement.

**Response:** We apologize for the clerical error in our previous submission regarding the ethical approval protocol number. We have verified the official documentation and confirm that the correct approval for this study is Protocol Number: **017/2024,** issued on **July 20, 2024,** by the Institutional Review Board of the College of Health Science, Debre Tabor University. We have updated the Ethics Statement in both the manuscript and the online submission system to reflect this correct protocol information. The official approval letter has been provided as a supplementary file for verification

Minor Issues

Language and grammar: The manuscript requires professional English editing (grammar, capitalization, tense consistency)

Abstract: Simplify methods and include clearer effect size reporting with confidence intervals

Terminology: Ensure consistent use of terms (e.g., HSS, SSS)

References: Revise for completeness and adherence to journal format

Formatting: Improve table titles, remove redundancy, and ensure consistency across sections

**Response-**

We look forward to receiving your revision

Reviewers' comments:

Reviewer #1: Reviewer Comments to the Authors and Author’s response

General Assessment

This manuscript addresses an important public health issue HPV-related knowledge, attitudes, and prevention practices among university students in Ethiopia using a comparative approach between health and social science disciplines. The topic is timely and relevant to global cervical cancer elimination strategies. The comparative design is a notable strength. However, several methodological inconsistencies, reporting issues, and interpretative concerns must be addressed before the manuscript can be considered for publication.

Major Comments

1. Study Design and Conceptual Clarity

- The manuscript inconsistently describes the study as “prospective” and “cross-sectional.” A cross-sectional study is, by definition in abstract and main manuscript, not prospective. Please revise throughout for consistency.

-The use of the Health Belief Model (HBM) is mentioned; however, it is not operationalized in the analysis. Variables corresponding to HBM constructs (e.g., perceived susceptibility, barriers) are not clearly measured or analyzed.

→ Recommendation: Either integrate HBM variables into the analysis or remove/reduce the theoretical claim.

**Response-** We thank the reviewer for identifying this inconsistency. We have removed the term "prospective" from the Abstract and the Methods section. The study is now consistently described as a "comparative cross-sectional study" throughout the manuscript.

Regarding Health belief model- Since our primary objective was to compare epidemiological determinants between the two student cohorts rather than to validate HBM constructs, we have removed the theoretical claims regarding the Health Belief Model from the Introduction and Methods sections. This ensures that our theoretical framework is fully aligned with our data analysis.

2. Sampling Procedure – Major Inconsistency

The manuscript states:

Use of multi-stage sampling and simple random sampling in abstract, but later reports convenience sampling.

This is a critical methodological contradiction that undermines internal validity.

→ Clarify:

- Exact sampling steps

- Whether probability sampling was truly implemented

- If convenience sampling was used, acknowledge selection bias explicitly.

**Response**- We have corrected the methodological contradiction identified by the reviewers. We have removed all references to 'multi-stage' and 'random' sampling. The study now consistently describes a quota-based convenience sampling approach (Methods, Lines 117 to 125). We have also added an explicit acknowledgment of selection bias as a study limitation (Limitations, Lines 461 to 468)

3. Outcome Definitions – Conceptual Issues

The definition of “practice” is problematic:

- Defined as completion of two-dose vaccination, yet later the limitation section states “at least one dose”.→ This inconsistency must be corrected.

- Using ≥50% cut-off for knowledge and attitude is arbitrary→ Provide justification or cite validated tools.

- Practice measured only via vaccination is too narrow→ Consider including screening behavior or intention.

**Response**- We have revised the "Operational Definitions" section (Lines 174–183) to address the conceptual concerns. Specifically, we have: Standardized the definition of Practice to "at least one dose" to ensure internal consistency.

Provided a technical justification for the 50% cut-off for knowledge and attitude, citing the modified Bloom’s criteria and relevant local literature [14].

Standardized the use of "good/poor" terminology across all KAP domains.

4. Data Availability – Not Compliant with Journal Policy

The statement “data available upon reasonable request” does not meet PLOS ONE requirements.

→ Authors must:

- Deposit data in a public repository, OR

- Provide a justified restriction (e.g., ethical/legal constraints with access mechanism).

**Response**- Thank you for this guidance regarding the PLOS ONE Data Availability Policy. We have updated our statement and our submission to ensure full compliance. We have now provided all relevant raw data as Supporting Information files.

These files include the fully de-identified datasets for both the Health Science and Social Science student cohorts, allowing for complete replication of our reported results, including the multivariable logistic regression and model diagnostics (VIF and Hosmer-Lemeshow tests).

5. Statistical Analysis – Insufficient Detail

The manuscript lacks key information:

- Variable selection criteria for multivariable models

- Multicollinearity assessment

- Model diagnostics (e.g., Hosmer-Lemeshow test)

Some reported findings are unstable (wide CIs), e.g.: AOR = 7.61 (1.65–35.12)

→ Interpret cautiously and acknowledge limited precision.

**Response**- We have comprehensively revised the statistical reporting to adhere to PLOS ONE’s standards: Variable Selection: We have specified that variables with p < 0.25 in bivariable analysis were entered into the multivariable model using backward stepwise elimination (Methods, Lines 215–226). Diagnostics: We now report Hosmer–Lemeshow test results, Nagelkerke R², and Classification Accuracy for all models. For the SSS knowledge model, we have justified its use despite the H-L result by highlighting its 80.3% predictive accuracy. Multicollinearity: We have confirmed model stability with VIF values for all predictors (HSS Max VIF = 1.836; SSS Max VIF = 1.252). Precision: We have added cautionary statements regarding variables with wide confidence intervals in both the Results and Limitations.

6. Results Section – Reporting Issues

In Table 2: Total respondents are incorrectly described as 335 instead of 670 in text.

- Source of information denominator (n=286) is unclear→ Explain missing data handling.

- Some tables: Lack clarity (e.g., Table 5 formatting, inconsistent totals), avoid redundancy between text and tables.

**Response-** We have corrected these reporting issues to ensure full transparency: Sample Size Consistency: We have updated the text and Table 2 to consistently reflect the total sample (N=670) and the equal distribution between the two study cohorts (n=335 each).

Denominator Clarification: We have clarified that the denominator for 'Source of Information' is derived only from participants with prior HPV awareness (n=470 total). We have added a specific footnote to Table 2 to explain this skip-logic, confirming that no data was missing, but rather that the question was only applicable to aware participants.

Table 5 Refinement: We have reformatted Table 5 for clarity and removed redundant descriptions from the text, as suggested.

7. Interpretation – Overstatement of Findings

The concept of “window of disengagement” is interesting but:

- Not directly measured

- Based on cross-sectional data → cannot infer temporal trends

Claims regarding: social media “misinformation” and Knowledge decay over time→ These are speculative and should be toned down.

**Response**- We have systematically toned down all causal and overreaching claims throughout the manuscript. First, we replaced causal language such as "knowledge decay" and "window of disengagement" with descriptive phrasing (e.g., "lower knowledge levels observed in later academic years") and added a disclaimer that these are hypotheses requiring longitudinal testing, as cross-sectional data cannot establish temporal trends. Second, we softened claims regarding Ethiopia's 90-70-90 targets: "requires" was changed to "could potentially contribute to," and we explicitly state that cross-sectional KAP studies cannot directly measure progress toward elimination targets. Third, we reframed speculation about misinformation as hypotheses (e.g., "may be associated with," "generates the hypothesis that") and added an explicit acknowledgment that we did not directly measure misinformation exposure. Finally, we added a new sentence to the Limitations section stating that all observed associations are correlational and causal inferences cannot be drawn. All changes are highlighted in the revised manuscript.

8. Discussion – Needs Better Balance

- The discussion is overly narrative and repetitive.

- Some comparisons lack critical appraisal of differences in study settings.

- Limited engagement with Ethiopian national HPV policy context, Vaccine availability and cost barriers

**Response**- We have revised the Discussion (Lines 406 to 418 and 426 to 429) to provide a rigorous contextualization of our vaccination rates using recent Ethiopian literature:

Regional Alignment: We noted that our results align with findings from Bahir Dar (16%), confirming the validity of our data in the Amhara region.

Target Group Disparity: We contrast our 18.2% rate with the 66.5% reported in primary schools (Kassa et al., 2021), explaining that university students fall into a "policy gap" as they are outside the primary target age for national school-based campaigns.

National Comparison: We have benchmarked our findings against the 2025 PMA national survey (30.8%) and the 2023 regional meta-analysis (41.46%), highlighting that university-aged women represent a underserved population in Ethiopia’s current vaccine rollout strategy.

Minor Comments

9. Language and Grammar

The manuscript requires extensive English editing:

- Capitalization errors (e.g., “Prevalent”, “Cervical Cancer”)

- Inconsistent tense usage

Suggest professional language editing.

**Response**- The manuscript has undergone extensive professional English editing. We have corrected capitalization errors and ensured consistent use of the past tense throughout the Methods and Results sections.

10. Abstract:

- Methods section is too dense

- Include clearer reporting of effect sizes with CI

**Response**- Abstract: The Methods section of the abstract has been simplified for better readability. All reported findings now include explicit Adjusted Odds Ratios (AOR) with their corresponding 95% Confidence Intervals (CI).

11. Terminology Consistency

- Ensure consistent use of:

“Health Science Students (HSS)”

“Social Science Students (SSS)”

Avoid switching terms throughout.

Response- We have standardized the terminology across the manuscript, consistently using "Health Science Students (HSS)" and "Social Science Students (SSS)."

12. Tables and Formatting

Improve:

- Table titles (more descriptive)

- Alignment and formatting

- Remove unnecessary duplication.

Response- Tables: Table titles have been revised to be more descriptive. We have improved the formatting and alignment of all tables and removed redundant data descriptions from the text.

13. References

Some references:

- Appear incomplete or inconsistently formatted

- Ensure adherence to journal style.

Response- : All references have been audited for completeness and reformatted to strictly adhere to the PLOS ONE (Vancouver) style.

Strengths of the Study

Large sample size (n=670

\# Comparative design across disciplines

\# Focus on an under-researched population in Ethiopia

\# Policy-relevant findings aligned with global target

Recommendation

Major Revision

The manuscript has strong potential but requires substantial methodological clarification, correction of inconsistencies, and improved analytical rigor before it can be considered for publication.

Reviewer #2: The manuscript addresses an important public-health topic (HPV knowledge, attitude, and prevention practices among university students). However, address the issues to improve the article standard.

1. The manuscript states: “prospective institutional-based comparative cross-sectional study”

This is methodologically incorrect terminology. A cross-sectional study cannot be prospective unless repeated measurement or follow-up exists.

**Response-** We thank the reviewer for identifying this inconsistency. We have removed the term "prospective" from the Abstract and the Methods section. The study is now consistently described as a "comparative cross-sectional study" throughout the manuscript.

2. In line 42, 43: Author claimed However, both groups

43 exhibited poor knowledge (HSS: 61.2%; SSS: 80.9%). How it was measured? is more than 60% poor?

Response: We appreciate the opportunity to clarify this. The percentages reported (HSS: 61.2%; SSS: 80.9%) refer to the proportion of students in each group who were categorized as having poor knowledge.

Measurement Criteria: "Poor knowledge" was defined as achieving a score of less than 50% on the knowledge-based questions (modified Bloom’s cut-off). Therefore, 61.2% of Health Science students failed to correctly answer at least half of the knowledge questions. We have clarified this definition in the Operational Definitions section (Line 172 to 175) to prevent further ambiguity.

3. Outcome variables categorized as:good/poor, cut-off criteria should have reference.

Response: We agree with the reviewer’s suggestion. We have updated the Operational Definitions section to include the scientific justification for the categorization of our outcome variables. Specifically, the 50% cut-off point used for knowledge and attitude scores follows the modified Bloom’s criteria, a standard threshold in similar epidemiological studies conducted in Ethiopia. We have added the following reference to support this:

Mekonnen BA, Anagaw YK, Kassahun BA, Worku MC. Evaluation of female university students’ knowledge, attitudes, and practices toward human papillomavirus infection and vaccination. Multicenter cross-sectional study. BMC Women's Health. 2024;24(1):437.

These changes can be found in the revised manuscript in the Methods section (Lines 172–185).

4. The study states:

selected via multistage sampling However: Missing: • sampling frame • stage-wise selection steps • proportional allocation method • response rate

Without these: Risk of selection bias

Response: We have revised the sampling section (Lines 117–125) to provide full methodological transparency.

Design Clarification: We have clarified that a quota-based convenience sampling approach was used instead of a probability-based multistage design, as a formal sampling frame (student roster) was unavailable.

Stage-wise Selection: We now describe the two-stage process: purposive college selection followed by proportional allocation across departments.

Response Rate: We have explicitly stated the response rate (100%) and explained the data collection window (June to July 2025).

Selection Bias: To address the reviewer's concern, we have also added a statement in the Limitations section acknowledging the risk of selection bias associated with this non-probability sampling method.

5. Regression model includes predictors but manuscript does not explain: whether variables entered based on p<0.25/p<0.20/p<0.05 screening • stepwise vs forced entry model. Standard epidemiologic reporting missing.

**Response**- We have revised the Data Processing and Analysis section (Lines 215–224) to provide the missing epidemiological details. We now explicitly state that: A p-value threshold of < 0.25 in the bivariable analysis was used for initial variable screening.

A backward stepwise elimination method was employed for the multivariable model building to identify independent predictors while controlling for confounders.

6. Multicollinearity assessment not reported. Expected reporting:VIF values ,tolerance values, Hosmer–Lemeshow test, pseudo-R² missing.

**Response-** We have updated the manuscript to include comprehensive diagnostic reporting for all multivariable logistic regression models. These additions ensure the statistical validity and reliability of our findings:

Multicollinearity: We have now reported the Variance Inflation Factor (VIF) values to confirm the absence of significant collinearity. All VIF values were well below the threshold of 10 (HSS Max VIF = 1.836; SSS Max VIF = 1.252).

Model Fitness: We have included the results of the Hosmer–Lemeshow test for each model to demonstrate adequate goodness-of-fit.

Explanatory Power: We have added the Nagelkerke R-square ($Pseudo-R^2$) and overall classification accuracy (e.g., 80.3% for the SSS knowledge model) to provide a clearer picture of the models' predictive utility.

6. In table 5, Why 5th year was used as reference?. It should be 1st. In table 6, 0.00 was reported in the academic year. It should be 0.001*

**Response**- As requested, we have recalculated the associations in Table 5 using the 2nd Year as the reference category. This adjustment provides a more intuitive interpretation of how students' attitudes shift as they progress through their academic program. While the odds of having a good attitude were higher in the 5th year compared to the 2nd year (COR = 2.46), the result remained statistically non-significant (p = 0.06). We have updated Table 5 and the corresponding text (Lines [Insert Line #]) to reflect these new values.

7. Vaccination rate discussion insufficient: Vaccination rates extremely low:• 18.2% • 15.2%

But manuscript does not discuss: vaccine availability , • national rollout status • cost barriers or policy context.

**Response** - We thank the reviewer for this insightful comment. We have significantly expanded the Discussion section (Lines [Insert Line #s]) to provide a multi-layered analysis of the low vaccination rates (15.2%–18.2%):

Policy Context: We now discuss the findings within the framework of Ethiopia’s National Cancer Control Plan (2025–2029) and the WHO "90-70-90" global targets.

Target Group Mismatch: We have clarified that Ethiopia’s National Expanded Program on Immunization primarily targets 9–14-year-olds. University students represent a "missed cohort" who are not prioritized in routine campaigns.

Systemic Barriers: We have added a discussion on logistical hurdles, including inconsistent supply, cold-chain requirements, and the financial barriers faced by young adults who must access the vaccine outside of school-based programs.

National Rollout Status: We have updated the text to reflect Ethiopia’s recent transition to a single-dose schedule and how actual uptake in higher education settings lags behind national goals for younger cohorts.

8. Literature comparison limited

Discussion lacks:• regional comparison (Africa / Asia) • LMIC HPV vaccination studies • university-level interventions evidence.

**Response-** We have significantly expanded the Discussion section (Lines 426 to 436) to provide regional and global context. Specifically, we have added comparisons with study findings from Nigeria (Reference 26), India (Reference 27), and Malaysia (Reference 28). These additions highlight that the low vaccination uptake and "catch-up gap" observed among our students are consistent with challenges faced by university populations across other LMICs in Africa and Asia.

9. Over-interpretation risk; Study claims implications for: achieving Ethiopia’s 90-70-90 targets However: Cross-sectional KAP studies cannot evaluate progress toward elimination targets. This should be softened.

**Response-** We agree with the reviewer that a cross-sectional study has inherent limitations in evaluating progress toward long-term global targets. We have revised the language in the Discussion and Conclusion (Lines 448 to 453) to "soften" these claims. We now state that our findings inform the challenges and identify barriers to reaching the 90-70-90 targets, rather than directly evaluating Ethiopia's progress toward them.
