# Supplementary material for: Knowledge, attitudes, and prevention practices regarding human papillomavirus among health and social science female students in Northwest Ethiopia: A comparative cross-sectional study
Source: PLoS One. 2026 Oct 5;21(10):e0359838. doi: 10.1371/journal.pone.0359838 (PMC13637910; doi:10.1371/journal.pone.0359838)
Supplement: Supplementary file 3 [file pone.0359838.s006.docx]

Author response

The authors express their sincere gratitude to the editor and reviewers for their insightful feedback and dedication to elevating the quality of this manuscript. Point-by-point responses to each of the editor's and reviewers' comments are detailed below, along with the corresponding revisions made to the manuscript.

**Reviewer 3 comments and Author responses**

1. Minor editing is needed for capitalization and consistency, particularly 'comparative cross‑sectional study.

**Author response**: We have corrected the capitalization in the title to read "Comparative Cross‑Sectional Study" as suggested.

2. In the abstract, the word 'prospective' should be removed because the study is cross‑sectional.

**Author response**: We have reviewed the abstract and the entire manuscript and confirm that the word "prospective" does not appear anywhere. The study design is consistently described as "cross‑sectional" throughout. No changes were needed for this comment.

3. Ensure that all abbreviations, including HSS and SSS, are defined at first use.

**Author Response:** We confirm that HSS (Health Science Students) and SSS (Social Science Students) are already defined at first use in the abstract (lines 42 and 43). No changes were required.

4. The conclusion should remain closely aligned with the observational findings and avoid implying that the proposed educational interventions were tested.

**Author response:** We have revised the conclusion to more clearly frame recommendations as implications of the observational findings, rather than implying that interventions were tested. The revised text now states: "Based on our observational findings, we recommend..."

5. Briefly clarify how the model informed questionnaire development or interpretation.

**Author response:** We have clarified in the Introduction (lines 77‑78) that the study was guided by a comparative empirical framework and did not impose a specific behavioral theory. The HBM was therefore not used to inform questionnaire development.

6. The term 'multicentre' should be replaced because the study was conducted at one university."

**Author response**: We have replaced the term "multicenter" with "comparative" in line 86, as the study was conducted at a single university (Debre Tabor University).

7. The final paragraph would benefit from a concise statement of the primary comparative objective and the expected difference between the two academic groups.

**Author response:** We have added a concise statement of the expected difference between groups to the final paragraph of the Introduction (lines 90‑92), stating that we hypothesized Health Science students would have higher knowledge and more favorable attitudes due to curriculum exposure.

8. Clarification is required regarding the sampling procedure because both simple random sampling and convenience sampling are mentioned.

**Author response**: We confirm that the sampling procedure is consistently described as "non‑probability quota‑based convenience sampling" and "purposive selection" of colleges. No mention of simple random sampling appears in the manuscript. No changes were required.

9. The questionnaire description should include the number of items in each domain, the possible score ranges, and reliability coefficients.

**Author response**: We thank the reviewer for this valuable suggestion. We have expanded the questionnaire description in the Methods section to specify the number of items and possible score ranges for each domain. Additionally, we have now calculated and reported Cronbach's alpha coefficients to assess internal consistency for both Health Science and Social Science student cohorts.

For the knowledge domain (14 items), Cronbach's alpha was 0.930 in both Health Science and Social Science students, indicating excellent internal consistency. For the attitude domain (5 items), Cronbach's alpha was 0.762 in both groups, indicating acceptable reliability. Corrected item-total correlations for all items exceeded 0.30, confirming that each item contributed meaningfully to its respective scale.

As the practice domain was measured as a single binary outcome (vaccination status: yes/no), Cronbach's alpha was not applicable for that domain. These reliability statistics have been added to the Data Quality Control section of the manuscript.

10. The 50% cut‑off used to define good and poor knowledge and attitude should be supported with a reference or brief justification.

**Author response:** We have expanded the justification for the 50% cut‑off in the Operational Definitions section (lines 154‑155) to reference modified Bloom's criteria and prior Ethiopian studies.

11. The definition of 'practice' is based primarily on completion of the HPV vaccination schedule. Consider using the more precise term 'vaccination practice' or 'HPV vaccine uptake'.

**Author response:** We have revised the terminology throughout the manuscript to use "vaccination practice" or "HPV vaccine uptake" where appropriate, and have updated Table 3 and the Abstract accordingly.

12. The wording regarding inter‑rater reliability should also be clarified because the questionnaire was self‑administered.

**Author response:** We have clarified that the inter‑rater reliability checks were conducted on the data abstraction process, not on the self‑administered questionnaire itself (lines 179).

13. The manuscript and submission form report different protocol numbers and approval dates. Verify and present consistently.

**Author response:** We have verified the ethics protocol number and approval date against the official approval letter. The manuscript now consistently reports protocol number 017/2024 (July 20, 2024), matching the submission form.

14. Add the relevant chi‑square test results and exact p‑values for comparisons between health and social science students.

**Author response:** We thank the reviewer for this valuable suggestion. To address this, we created a grouping variable (academic_discipline) in both datasets before merging (1 = Health Science, 2 = Social Science). Using the combined dataset (N=670), we performed Pearson's chi-square tests to formally compare the two groups across all key outcomes. The results, now added to the Results section (lines 240-245), show:

HPV knowledge (good knowledge): χ² = 31.61, df = 1, p < 0.001

Attitude towards HPV vaccination (good attitude): χ² = 26.07, df = 1, p < 0.001

Vaccination practice (good practice): χ² = 1.07, df = 1, p = 0.300

These formal comparisons confirm that Health Science students have significantly higher knowledge and more favorable attitudes compared to Social Science students, while vaccination practice does not differ significantly between the two groups. The non-significant practice finding aligns with the low overall vaccination rates observed in both groups (18.2% vs. 15.2%).

15. In the regression tables, p = 0.00 should be reported as p < .001.

**Author Response**: All p‑values previously reported as "0.00" have been corrected to "<0.001" in accordance with standard reporting guidelines.

16. Wide confidence intervals should be acknowledged as indicating imprecision.

**Author Response:** We have already acknowledged the wide confidence intervals in the Results section (lines 257‑260) and noted that these estimates should be interpreted with caution. No additional changes were required.

17. The denominators used for the sources‑of‑information percentages in Table 2 should be checked and clearly stated for each academic group.

**Author Response:** We confirm that the denominators for the sources‑of‑information percentages in Table 2 are already clearly stated in the footnote (n=280 for HSS and n=190 for SSS). No changes were required.

18. Clearly distinguish awareness, knowledge, attitudes, and vaccination practice.

**Author Response:** We confirm that awareness, knowledge, attitudes, and vaccination practice are already clearly defined and distinguished in the Operational Definitions section (lines 150‑162) and are used consistently throughout the manuscript. No changes were required.

19. Explanations related to curriculum exposure, social media use, access, and vaccine availability should be framed as possible interpretations rather than confirmed causes.

**Author Response:** We have reviewed the Discussion and replaced definitive causal language with more cautious phrasing, using qualifiers such as "may," "likely," and "suggests" to frame explanations as possible interpretations rather than confirmed causes.

**Reviewer 4 comments and author responses.**

1. The Methods section contains remnants of the original multi‑stage sampling description. Remove all remaining references to multi‑stage and random sampling.

Author Response: We have reviewed the Sampling section and confirm that it consistently describes "non‑probability quota‑based convenience sampling" and "purposive selection" of colleges. No references to multi‑stage or random sampling remain in the manuscript. No changes were required for this comment.

2. The Data Availability Statement is not compliant with PLOS ONE policy. Update to indicate data are included as Supporting Information files.

**Author Response:** We confirm that the Data Availability Statement already states that all relevant data are included as Supporting Information files (Line 528-530 - All relevant data supporting the conclusions of this study are included as Supporting Information files (S1 Data: Health Science Students and S2 Data: Social Science Students), in compliance with PLOS ONE policy. No changes were required.

3. Tables 4, 5, and 6 are poorly formatted with malformed numerical entries. Correct all

**Author Response:** We have carefully reviewed Tables 4, 5, and 6 and corrected any formatting inconsistencies. All Adjusted Odds Ratios are now reported to two decimal places, confidence intervals are formatted consistently, and p‑values are presented as <0.001 where appropriate. Footnotes explaining abbreviations have been added.

4. The phrase 'window of disengagement' still appears in the Discussion. Remove it completely.

**Author Response**: We have removed the phrase "window of disengagement" from the manuscript (line 94) and replaced it with descriptive wording. We have also corrected the duplicate phrase in line 366 to "creating an observed gap in later academic years.

5. Table 6 still contains '0.00' – change to '<0.001'. Also ensure all p‑values are consistent.

**Author Response:** We have reviewed all regression tables and confirmed that p‑values are now consistently reported as <0.001 where appropriate. No instances of 0.00 or 0.000 remain.

6. Add a brief explanation for using 2nd year as the reference category in Table 5.

**Author Response**: We have added a brief explanation in the Statistical Analysis section (lines 284‑286) justifying the use of 2nd year as the reference category for academic year in Table 5. "For multivariable logistic regression, the reference category for academic year was set to 2nd year to provide a more intuitive interpretation of changes in attitudes and practices as students’ progress through their academic program."

7. The abstract contains a duplicate Methods section. Remove the duplicate.

**Author response:** We have reviewed the Abstract and confirm that there is no duplicate Methods section. The abstract follows a standard structure (Background, Objective, Methods, Results, Conclusion). No changes were required.

8. Standardize capitalization of 'Social Science' throughout.

**Author response:** We have standardized the capitalization of "Social Science" throughout the manuscript to ensure consistency.

9. Add actual denominator values (n=280 for HSS, n=190 for SSS) to Table 2 footnote.

**Author response:** We confirm that the denominators (n=280 for HSS and n=190 for SSS) are already clearly stated in the Table 2 footnote (Note: The percentages for 'Source of Information' are calculated based on the subset of participants who reported prior awareness of HPV (n=280 for Health Science and n=190 for Social Science). This explains the variation in denominators compared to the total sample size (N=670). No changes were required.
