# Supplementary material for: Knowledge, attitudes, and prevention practices regarding human papillomavirus among health and social science female students in Northwest Ethiopia: A comparative cross-sectional study
Source: PLoS One. 2026 Oct 5;21(10):e0359838. doi: 10.1371/journal.pone.0359838 (PMC13637910; doi:10.1371/journal.pone.0359838)
Supplement: Peer review history

# Peer review: Decision Letter 0

**DOI:** 10.1371/journal.pone.0359838.r001

**Authors:** Abera Lambebo Temamo

14 Apr 2026

Dear Dr. malkamu,

Thank you for submitting your manuscript to PLOS ONE. After careful consideration, we feel that it has merit but does not fully meet PLOS ONE’s publication criteria as it currently stands. Therefore, we invite you to submit a revised version of the manuscript that addresses the points raised during the review process.

Please submit your revised manuscript by May 29 2026 11:59PM. If you will need more time than this to complete your revisions, please reply to this message or contact the journal office at plosone@plos.org. When you're ready to submit your revision, log on to https://www.editorialmanager.com/pone/ and select the 'Submissions Needing Revision' folder to locate your manuscript file.

- A letter that responds to each point raised by the academic editor and reviewer(s). You should upload this letter as a separate file labeled 'Response to Reviewers'.
- A marked-up copy of your manuscript that highlights changes made to the original version. You should upload this as a separate file labeled 'Revised Manuscript with Track Changes'.
- An unmarked version of your revised paper without tracked changes. You should upload this as a separate file labeled 'Manuscript'.

If you would like to make changes to your financial disclosure, please include your updated statement in your cover letter. Guidelines for resubmitting your figure files are available below the reviewer comments at the end of this letter.

If applicable, we recommend that you deposit your laboratory protocols in protocols.io to enhance the reproducibility of your results. Protocols.io assigns your protocol its own identifier (DOI) so that it can be cited independently in the future. For instructions see: https://journals.plos.org/plosone/s/submission-guidelines#loc-laboratory-protocols. Additionally, PLOS ONE offers an option for publishing peer-reviewed Lab Protocol articles, which describe protocols hosted on protocols.io. Read more information on sharing protocols at https://plos.org/protocols?utm_medium=editorial-email&utm_source=authorletters&utm_campaign=protocols.

As the corresponding author, your ORCID iD is verified in the submission system and will appear in the published article. PLOS supports the use of ORCID, and we encourage all coauthors to register for an ORCID iD and use it as well. Please encourage your coauthors to verify their ORCID iD within the submission system before final acceptance, as unverified ORCID iDs will not appear in the published article. *Only*  the individual author can complete the verification step; PLOS staff *cannot*  verify ORCID iDs on behalf of authors.

We look forward to receiving your revised manuscript.

Kind regards,

Abera Lambebo Temamo, Ph.D.

Academic Editor

PLOS One

Journal Requirements:

When submitting your revision, we need you to address these additional requirements.

1.Please ensure that your manuscript meets PLOS ONE's style requirements, including those for file naming. The PLOS ONE style templates can be found at

https://journals.plos.org/plosone/s/file?id=wjVg/PLOSOne_formatting_sample_main_body.pdf and

https://journals.plos.org/plosone/s/file?id=ba62/PLOSOne_formatting_sample_title_authors_affiliations.pdf

2. Please provide additional details regarding participant consent. In the ethics statement in the Methods and online submission information, please ensure that you have specified (1) whether consent was informed and (2) what type you obtained (for instance, written or verbal, and if verbal, how it was documented and witnessed). If your study included minors, state whether you obtained consent from parents or guardians. If the need for consent was waived by the ethics committee, please include this information.

If you are reporting a retrospective study of medical records or archived samples, please ensure that you have discussed whether all data were fully anonymized before you accessed them and/or whether the IRB or ethics committee waived the requirement for informed consent. If patients provided informed written consent to have data from their medical records used in research, please include this information.

3. The ethical approval number(s) listed in the submission metadata does not match the approval number on the ethical approval document you provided. Please ensure that all approval numbers are correct.

4. In the online submission form, you indicated that “data are available upon reasonable request”.

All PLOS journals now require all data underlying the findings described in their manuscript to be freely available to other researchers, either 1. In a public repository, 2. Within the manuscript itself, or 3. Uploaded as supplementary information.

This policy applies to all data except where public deposition would breach compliance with the protocol approved by your research ethics board. If your data cannot be made publicly available for ethical or legal reasons (e.g., public availability would compromise patient privacy), please explain your reasons on resubmission and your exemption request will be escalated for approval.

5. Your ethics statement should only appear in the Methods section of your manuscript. If your ethics statement is written in any section besides the Methods, please delete it from any other section.

6. If the reviewer comments include a recommendation to cite specific previously published works, please review and evaluate these publications to determine whether they are relevant and should be cited. There is no requirement to cite these works unless the editor has indicated otherwise.

Additional Editor Comments:

Dear Authors,

Thank you for submitting your manuscript examining HPV-related knowledge, attitudes, and prevention practices among university students in Ethiopia. Both reviewers recognize that your study addresses an important and policy-relevant public health issue, particularly within the context of cervical cancer prevention and global elimination efforts. The comparative approach between health science and social science students, as well as the relatively large sample size, are notable strengths.

However, after careful evaluation of the reviewers’ comments, I must inform you that the manuscript requires major revision before it can be considered further for publication. There are substantial concerns regarding methodological clarity, analytical rigor, and interpretation of findings that need to be addressed comprehensively.

Below, I synthesize the key issues raised by both reviewers to guide your revision.

Major Issues Requiring Revision

1. Study Design and Conceptual Clarity

The manuscript repeatedly describes the study as a “prospective cross-sectional” design, which is methodologically incorrect. A cross-sectional study is not prospective unless repeated measures or follow-up are involved.

Please revise this terminology consistently throughout the manuscript.

The Health Belief Model (HBM) is mentioned but not clearly operationalized in the analysis.

Either incorporate measurable HBM constructs (e.g., perceived susceptibility, barriers) into the analysis or remove/reduce this theoretical framing.

2. Sampling Procedure and Risk of Bias

There are critical inconsistencies and missing details regarding sampling:

Contradiction between multi-stage/random sampling and convenience sampling

Missing essential elements:

Sampling frame

Stage-wise selection procedures

Proportional allocation

Response rate

These gaps raise concerns about selection bias and internal validity.

You must:

Clearly describe the exact sampling procedure step-by-step

Resolve inconsistencies across sections

Explicitly acknowledge limitations if non-probability sampling was used

3. Outcome Definitions and Measurement

Several conceptual and operational issues were identified:

The classification of knowledge and attitude (e.g., ≥50% cut-off) appears arbitrary

Provide justification or cite validated instruments

The statement that >60% reflects “poor knowledge” requires clarification and justification

The definition of “practice” is inconsistent:

Defined as full vaccination (two doses) in one section

Later described as at least one dose

Measuring practice solely through vaccination is too narrow

Consider including screening behavior or intention if data are available

4. Statistical Analysis and Reporting

The manuscript lacks key epidemiological reporting standards:

No clear explanation of:

Variable selection criteria (e.g., p-value thresholds such as p<0.25)

Model-building strategy (stepwise vs. forced entry)

Missing diagnostics:

Multicollinearity assessment (e.g., VIF, tolerance)

Model fit (e.g., Hosmer–Lemeshow test, pseudo R²)

Additionally:

Some estimates show wide confidence intervals, indicating instability

Interpret cautiously and acknowledge limited precision

5. Results Presentation

Inconsistencies in reported sample size (e.g., 335 vs. 670)

Unclear denominators (e.g., source of information n=286)

Table issues:

Poor formatting and unclear structure (e.g., Table 5)

Inappropriate reference category (e.g., use of 5th year instead of 1st year)

Incorrect p-value reporting (e.g., “0.00” should be reported as “<0.001”)

Revise all tables for clarity, accuracy, and consistency with journal standards.

6. Interpretation and Overstatement of Findings

The manuscript makes causal and temporal interpretations (e.g., knowledge decay, “window of disengagement”) that are not supported by cross-sectional data

Claims regarding contribution to Ethiopia’s 90–70–90 targets should be substantially toned down

Statements about misinformation and behavioral trends are speculative and should be clearly framed as such or removed

7. Discussion – Depth and Context

The discussion requires strengthening:

Expand literature comparison, especially:

Studies from Africa and other LMICs

University-based HPV interventions

Provide contextual discussion of:

HPV vaccine availability in Ethiopia

National rollout status

Cost and policy barriers

Current vaccination rates (18.2% and 15.2%) are very low but insufficiently contextualized

8. Data Availability (PLOS ONE Policy Compliance)

The statement “data available upon reasonable request” does not comply with PLOS ONE policy

You must:

Deposit data in a public repository, OR

Provide a clear and justified restriction with an access mechanism

Minor Issues

Language and grammar: The manuscript requires professional English editing (grammar, capitalization, tense consistency)

Abstract: Simplify methods and include clearer effect size reporting with confidence intervals

Terminology: Ensure consistent use of terms (e.g., HSS, SSS)

References: Revise for completeness and adherence to journal format

Formatting: Improve table titles, remove redundancy, and ensure consistency across sections

We look forward to receiving your revision

[Note: HTML markup is below. Please do not edit.]

Reviewers' comments:

Reviewer's Responses to Questions

**Comments to the Author**

1. Is the manuscript technically sound, and do the data support the conclusions?

Reviewer #1: Yes

Reviewer #2: Yes

**********

2. Has the statistical analysis been performed appropriately and rigorously?

Reviewer #1: Yes

Reviewer #2: No

**********

3. Have the authors made all data underlying the findings in their manuscript fully available?

The PLOS Data policy

Reviewer #1: Yes

Reviewer #2: No

**********

4. Is the manuscript presented in an intelligible fashion and written in standard English?

Reviewer #1: No

Reviewer #2: Yes

**********

Reviewer #1: Reviewer Comments to the Authors

General Assessment

This manuscript addresses an important public health issue HPV-related knowledge, attitudes, and prevention practices among university students in Ethiopia using a comparative approach between health and social science disciplines. The topic is timely and relevant to global cervical cancer elimination strategies. The comparative design is a notable strength. However, several methodological inconsistencies, reporting issues, and interpretative concerns must be addressed before the manuscript can be considered for publication.

Major Comments

1. Study Design and Conceptual Clarity

- The manuscript inconsistently describes the study as “prospective” and “cross-sectional.” A cross-sectional study is, by definition in abstract and main manuscript, not prospective. Please revise throughout for consistency.

-The use of the Health Belief Model (HBM) is mentioned; however, it is not operationalized in the analysis. Variables corresponding to HBM constructs (e.g., perceived susceptibility, barriers) are not clearly measured or analyzed.

→ Recommendation: Either integrate HBM variables into the analysis or remove/reduce the theoretical claim.

2. Sampling Procedure – Major Inconsistency

The manuscript states:

Use of multi-stage sampling and simple random sampling in abstract, but later reports convenience sampling.

This is a critical methodological contradiction that undermines internal validity.

→ Clarify:

- Exact sampling steps

- Whether probability sampling was truly implemented

- If convenience sampling was used, acknowledge selection bias explicitly.

3. Outcome Definitions – Conceptual Issues

The definition of “practice” is problematic:

- Defined as completion of two-dose vaccination, yet later the limitation section states “at least one dose”.→ This inconsistency must be corrected.

- Using ≥50% cut-off for knowledge and attitude is arbitrary→ Provide justification or cite validated tools.

- Practice measured only via vaccination is too narrow→ Consider including screening behavior or intention.

4. Data Availability – Not Compliant with Journal Policy

The statement “data available upon reasonable request” does not meet PLOS ONE requirements.

→ Authors must:

- Deposit data in a public repository, OR

- Provide a justified restriction (e.g., ethical/legal constraints with access mechanism).

5. Statistical Analysis – Insufficient Detail

The manuscript lacks key information:

- Variable selection criteria for multivariable models

- Multicollinearity assessment

- Model diagnostics (e.g., Hosmer-Lemeshow test)

Some reported findings are unstable (wide CIs), e.g.: AOR = 7.61 (1.65–35.12)

→ Interpret cautiously and acknowledge limited precision.

6. Results Section – Reporting Issues

In Table 2: Total respondents are incorrectly described as 335 instead of 670 in text.

- Source of information denominator (n=286) is unclear→ Explain missing data handling.

- Some tables: Lack clarity (e.g., Table 5 formatting, inconsistent totals), avoid redundancy between text and tables.

7. Interpretation – Overstatement of Findings

The concept of “window of disengagement” is interesting but:

- Not directly measured

- Based on cross-sectional data → cannot infer temporal trends

Claims regarding: Social media “misinformation” and Knowledge decay over time→ These are speculative and should be toned down.

8. Discussion – Needs Better Balance

- The discussion is overly narrative and repetitive.

- Some comparisons lack critical appraisal of differences in study settings.

- Limited engagement with Ethiopian national HPV policy context, Vaccine availability and cost barriers

Minor Comments

9. Language and Grammar

The manuscript requires extensive English editing:

- Capitalization errors (e.g., “Prevalent”, “Cervical Cancer”)

- Inconsistent tense usage

Suggest professional language editing.

10. Abstract:

- Methods section is too dense

- Include clearer reporting of effect sizes with CI

11. Terminology Consistency

- Ensure consistent use of:

“Health Science Students (HSS)”

“Social Science Students (SSS)”

Avoid switching terms throughout.

12. Tables and Formatting

Improve:

- Table titles (more descriptive)

- Alignment and formatting

- Remove unnecessary duplication.

13. References

Some references:

- Appear incomplete or inconsistently formatted

- Ensure adherence to journal style.

Strengths of the Study

Large sample size (n=670

# Comparative design across disciplines

# Focus on an under-researched population in Ethiopia

# Policy-relevant findings aligned with global target

Recommendation

Major Revision

The manuscript has strong potential but requires substantial methodological clarification, correction of inconsistencies, and improved analytical rigor before it can be considered for publication.

Reviewer #2: The manuscript addresses an important public-health topic (HPV knowledge, attitude, and prevention practices among university students).However, address the issues to improve the article standard.

1. The manuscript states:“prospective institutional-based comparative cross-sectional study”

This is methodologically incorrect terminology. A cross-sectional study cannot be prospective unless repeated measurement or follow-up exists.

2. In line 42, 43: Author claimed However, both groups

43 exhibited poor knowledge (HSS: 61.2%; SSS: 80.9%). How it was measured? is more than 60% poor?

3. Outcome variables categorized as:good/poor, cut-off criteria should have reference.

4. The study states:

selected via multistage sampling However:Missing: • sampling frame • stage-wise selection steps • proportional allocation method • response rate

Without these: Risk of selection bias

5. Regression model includes predictors but manuscript does not explain: whether variables entered based on p<0.25/p<0.20/p<0.05 screening • stepwise vs forced entry model. Standard epidemiologic reporting missing.

6. Multicollinearity assessment not reported. Expected reporting:VIF values ,tolerance values, Hosmer–Lemeshow test, pseudo-R² missing.

6. In table 5, Why 5th year was used as reference?. It should be 1st. In table 6, 0.00 was reported in the academic year. It should be 0.001*

7. Vaccination rate discussion insufficient: Vaccination rates extremely low:• 18.2% • 15.2%

But manuscript does not discuss:vaccine availability , • national rollout status • cost barriers or policy context

8. Literature comparison limited

Discussion lacks:• regional comparison (Africa / Asia) • LMIC HPV vaccination studies • university-level interventions evidence

9. Over-interpretation risk;Study claims implications for:achieving Ethiopia’s 90-70-90 targets

However:Cross-sectional KAP studies cannot evaluate progress toward elimination targets. This should be softened.

**********

what does this mean?). If published, this will include your full peer review and any attached files.

If you choose “no”, your identity will remain anonymous but your review may still be made public.

**Do you want your identity to be public for this peer review?**  For information about this choice, including consent withdrawal, please see our Privacy Policy

Reviewer #1: No

Reviewer #2: **Yes:**  Mohammad Injamul Hoq

**********

[NOTE: If reviewer comments were submitted as an attachment file, they will be attached to this email and accessible via the submission site. Please log into your account, locate the manuscript record, and check for the action link "View Attachments". If this link does not appear, there are no attachment files.]

To ensure your figures meet our technical requirements, please review our figure guidelines: https://journals.plos.org/plosone/s/figures

You may also use PLOS’s free figure tool, NAAS, to help you prepare publication quality figures: https://journals.plos.org/plosone/s/figures#loc-tools-for-figure-preparation.

NAAS will assess whether your figures meet our technical requirements by comparing each figure against our figure specifications.

# Author comment: Author response to Decision Letter 1

**DOI:** 10.1371/journal.pone.0359838.r002

18 May 2026

Dear Editor and Reviewers, on behalf of the authoring team, I would like to express our sincere gratitude for the constructive feedback provided during the initial review of our manuscript. In this revised version, we have not only addressed each of the reviewers' specific comments but have also undertaken a comprehensive internal audit to enhance the transparency, statistical robustness, and methodological clarity of our findings.

Major Issues Requiring Revision

1. Study Design and Conceptual Clarity

The manuscript repeatedly describes the study as a “prospective cross-sectional” design, which is methodologically incorrect. A cross-sectional study is not prospective unless repeated measures or follow-up are involved.

Response- the authors agree on the above comment; we removed the term ‘prospective’

Please revise this terminology consistently throughout the manuscript.

The Health Belief Model (HBM) is mentioned but not clearly operationalized in the analysis.

Either incorporate measurable HBM constructs (e.g., perceived susceptibility, barriers) into the analysis or remove/reduce this theoretical framing.

Response- We have removed the reference to the Health Belief Model, as our survey did not directly operationalize its constructs. The manuscript now presents the findings without theoretical framing, which we believe improves clarity and honesty regarding our data's limitations

2. Sampling Procedure and Risk of Bias

There are critical inconsistencies and missing details regarding sampling:

Contradiction between multi-stage/random sampling and convenience sampling

Missing essential elements:

Sampling frame

Stage-wise selection procedures

Proportional allocation

Response rate

These gaps raise concerns about selection bias and internal validity.

You must:

Clearly describe the exact sampling procedure step-by-step

Resolve inconsistencies across sections

Explicitly acknowledge limitations if non-probability sampling was used

Response- We have revised the Sampling section to accurately reflect the use of convenience sampling due to the lack of a real-time sampling frame, resolving the previous contradiction between random and convenience methods. We have clarified the participant flow in the Data collection section line 161 to 164. We employed a quota-based recruitment strategy where data collection was maintained within each academic cohort until the pre-calculated sample size of 335 per group (Total N=670) was reached. This ensured the study achieved 100% of its intended sample size to maintain statistical power. We have also updated the Limitations section line 444 to 451 to explicitly acknowledge the selection bias inherent in convenience sampling and how it relates to the internal validity of our findings.

3. Outcome Definitions and Measurement

Several conceptual and operational issues were identified:

The classification of knowledge and attitude (e.g., ≥50% cut-off) appears arbitrary

Provide justification or cite validated instruments

The statement that >60% reflects “poor knowledge” requires clarification and justification

The definition of “practice” is inconsistent:

Defined as full vaccination (two doses) in one section

Later described as at least one dose

Measuring practice solely through vaccination is too narrow

Consider including screening behavior or intention if data are available.

Response- We have addressed these conceptual concerns as follows:

Standardized Definition: We have standardized the definition of "Practice" as receiving at least one dose of the HPV vaccine. This definition is now used consistently across the Methods (Line 177to 179), Results, and Discussion.

Acknowledging Scope: We agree that including screening and intention data would have strengthened the study. Since this data was not collected, we have explicitly acknowledged this as a limitation (Line 414 to 417).

Future Recommendations: We have added a recommendation (Line 421-423) for future research to include screening behaviors and behavioral intentions to provide a more comprehensive view of HPV prevention.

4. Statistical Analysis and Reporting

The manuscript lacks key epidemiological reporting standards:

No clear explanation of:

Variable selection criteria (e.g., p-value thresholds such as p<0.25)

Model-building strategy (stepwise vs. forced entry)

Missing diagnostics:

Multicollinearity assessment (e.g., VIF, tolerance)

Model fit (e.g., Hosmer–Lemeshow test, pseudo R²)

Additionally:

Some estimates show wide confidence intervals, indicating instability

Interpret cautiously and acknowledge limited precision

Response- We have comprehensively revised the statistical reporting to adhere to PLOS ONE’s standards: Variable Selection: We have specified that variables with p < 0.25 in bivariable analysis were entered into the multivariable model using backward stepwise elimination (Methods, Lines 215–226). Diagnostics: We now report Hosmer–Lemeshow test results, Nagelkerke R², and Classification Accuracy for all models. For the SSS knowledge model, we have justified its use despite the H-L result by highlighting its 80.3% predictive accuracy. Multicollinearity: We have confirmed model stability with VIF values for all predictors (HSS Max VIF = 1.836; SSS Max VIF = 1.252). Precision: We have added cautionary statements regarding variables with wide confidence intervals in both the Results and Limitations.

5. Results Presentation

Inconsistencies in reported sample size (e.g., 335 vs. 670)

Unclear denominators (e.g., source of information n=286)

Table issues:

Poor formatting and unclear structure (e.g., Table 5)

Inappropriate reference category (e.g., use of 5th year instead of 1st year)

Incorrect p-value reporting (e.g., “0.00” should be reported as “<0.001”)

Revise all tables for clarity, accuracy, and consistency with journal standards.

Response- We thank the editor for the constructive feedback regarding our statistical reporting. We have revised the Data Processing and Analysis section (Lines 215 to 224) and the Results section (Lines 262 to 269 and 288 to 289) to incorporate these key epidemiological standards:

Variable Selection and Model Building: We have clarified that variables with a p-value < 0.25 in the bivariable analysis were selected as candidates for the multivariable logistic regression. A backward stepwise elimination method was employed to identify independent predictors and control for potential confounders.

Multicollinearity Assessment: We performed diagnostic checks for multicollinearity using the Variance Inflation Factor (VIF). All values were well below the threshold of 10 (Health Science VIF range: 1.02–1.84; Social Science VIF range: 1.01–1.25), confirming the independence of the predictors.

Model Fit and Diagnostics: We have now reported the Hosmer–Lemeshow (H-L) test and Nagelkerke R Square for all multivariable models to demonstrate model fitness:

Health Science Cohort: The models showed adequate fit for both Knowledge (p = 0.336) and Practice (p = 0.066).

Social Science Cohort: The Practice model showed an excellent fit (p = 0.858). While the Knowledge model yielded a significant H-L result (p = 0.001), we confirmed its predictive utility through a high overall classification accuracy of 80.3% and a Nagelkerke R Square of 0.237.

Interpretation of Confidence Intervals: We acknowledge that some variables, such as 'Heard about HPV' in Table 4, exhibited wide 95% Confidence Intervals (CI: 1.65–35.12). We have added a cautionary statement in the Results (Lines 262 to 265) and Limitations sections (Lines 449 to 453) acknowledging that these specific estimates have limited precision and should be interpreted with caution.

Refined P-value Reporting: All p-values previously reported as "0.000" have been corrected to < 0.001 throughout the manuscript and tables.

Sample Size Consistency: We have updated the text and Table 2 to consistently reflect the total sample (N=670) and the equal distribution between the two study cohorts (n=335 each).

Denominator Clarification: We have clarified that the denominator for 'Source of Information' is derived only from participants with prior HPV awareness (n=470 total). We have added a specific footnote to Table 2 to explain this skip-logic, confirming that no data was missing, but rather that the question was only applicable to aware participants.

Table 5 Refinement: We have reformatted Table 5 for clarity and removed redundant descriptions from the text, as suggested.

6. Interpretation and Overstatement of Findings

The manuscript makes causal and temporal interpretations (e.g., knowledge decay, “window of disengagement”) that are not supported by cross-sectional data

Claims regarding contribution to Ethiopia’s 90–70–90 targets should be substantially toned down

Statements about misinformation and behavioral trends are speculative and should be clearly framed as such or removed

Response- We have systematically toned down all causal and overreaching claims throughout the manuscript. First, we replaced causal language such as "knowledge decay" and "window of disengagement" with descriptive phrasing (e.g., "lower knowledge levels observed in later academic years") and added a disclaimer that these are hypotheses requiring longitudinal testing, as cross-sectional data cannot establish temporal trends. Second, we softened claims regarding Ethiopia's 90-70-90 targets: "requires" was changed to "could potentially contribute to," and we explicitly state that cross-sectional KAP studies cannot directly measure progress toward elimination targets. Third, we reframed speculation about misinformation as hypotheses (e.g., "may be associated with," "generates the hypothesis that") and added an explicit acknowledgment that we did not directly measure misinformation exposure. Finally, we added a new sentence to the Limitations section stating that all observed associations are correlational and causal inferences cannot be drawn. All changes are highlighted in the revised manuscript.

7. Discussion – Depth and Context The discussion requires strengthening:

Expand literature comparison, especially: Studies from Africa and other LMICs University-based HPV interventions

Provide contextual discussion of: HPV vaccine availability in Ethiopia

National rollout status

Cost and policy barriers

Current vaccination rates (18.2% and 15.2%) are very low but insufficiently contextualized

Response- We have revised the Discussion (Lines 406 to 418 and 426 to 429) to provide a rigorous contextualization of our vaccination rates using recent Ethiopian literature:

Regional Alignment: We noted that our results align with findings from Bahir Dar (16%), confirming the validity of our data in the Amhara region.

Target Group Disparity: We contrast our 18.2% rate with the 66.5% reported in primary schools (Kassa et al., 2021), explaining that university students fall into a "policy gap" as they are outside the primary target age for national school-based campaigns.

National Comparison: We have benchmarked our findings against the 2025 PMA national survey (30.8%) and the 2023 regional meta-analysis (41.46%), highlighting that university-aged women represent a underserved population in Ethiopia’s current vaccine rollout strategy.

8. Data Availability (PLOS ONE Policy Compliance)

The statement “data available upon reasonable request” does not comply with PLOS ONE policy

You must:

Deposit data in a public repository, OR

Provide a clear and justified restriction with an access mechanism

Response- Thank you for this guidance regarding the PLOS ONE Data Availability Policy. We have updated our statement and our submission to ensure full compliance. We have now provided all relevant raw data as Supporting Information files.

These files include the fully de-identified datasets for both the Health Science and Social Science student cohorts, allowing for complete replication of our reported results, including the multivariable logistic regression and model diagnostics (VIF and Hosmer-Lemeshow tests).

Minor Issues

Language and grammar: The manuscript requires professional English editing (grammar, capitalization, tense consistency)

Abstract: Simplify methods and include clearer effect size reporting with confidence intervals

Terminology: Ensure consistent use of terms (e.g., HSS, SSS)

References: Revise for completeness and adherence to journal format

Formatting: Improve table titles, remove redundancy, and ensure consistency across sections

Response-

We look forward to receiving your revision

Reviewers' comments:

Reviewer #1: Reviewer Comments to the Authors and Author’s response

General Assessment

This manuscript addresses an important public health issue HPV-related knowledge, attitudes, and prevention practices among university students in Ethiopia using a comparative approach between health and social science disciplines. The topic is timely and relevant to global cervical cancer elimination strategies. The comparative design is a notable strength. However, several methodological inconsistencies, reporting issues, and interpretative concerns must be addressed before the manuscript can be considered for publication.

Major Comments

1. Study Design and Conceptual Clarity

- The manuscript inconsistently describes the study as “prospective” and “cross-sectional.” A cross-sectional study is, by definition in abstract and main manuscript, not prospective. Please revise throughout for consistency.

-The use of the Health Belief Model (HBM) is mentioned; however, it is not operationalized in the analysis. Variables corresponding to HBM constructs (e.g., perceived susceptibility, barriers) are not clearly measured or analyzed.

→ Recommendation: Either integrate HBM variables into the analysis or remove/reduce the theoretical claim.

Response- We thank the reviewer for identifying this inconsistency. We have removed the term "prospective" from the Abstract and the Methods section. The study is now consistently described as a "comparative cross-sectional study" throughout the manuscript.

Regarding Health belief model- Since our primary objective was to compare epidemiological determinants between the two student cohorts rather than to validate HBM constructs, we have removed the theoretical claims regarding the Health Belief Model from the Introduction and Methods sections. This ensures that our theoretical framework is fully aligned with our data analysis.

2. Sampling Procedure – Major Inconsistency

The manuscript states:

Use of multi-stage sampling and simple random sampling in abstract, but later reports convenience sampling.

This is a critical methodological contradiction that undermines internal validity.

→ Clarify:

- Exact sampling steps

- Whether probability sampling was truly implemented

- If convenience sampling was used, acknowledge selection bias explicitly.

Response- We have corrected the methodological contradiction identified by the reviewers. We have removed all references to 'multi-stage' and 'random' sampling. The study now consistently describes a quota-based convenience sampling approach (Methods, Lines 117 to 125). We have also added an explicit acknowledgment of selection bias as a study limitation (Limitations, Lines 461 to 468)

3. Outcome Definitions – Conceptual Issues

The definition of “practice” is problematic:

- Defined as completion of two-dose vaccination, yet later the limitation section states “at least one dose”.→ This inconsistency must be corrected.

- Using ≥50% cut-off for knowledge and attitude is arbitrary→ Provide justification or cite validated tools.

- Practice measured only via vaccination is too narrow→ Consider including screening behavior or intention.

Response- We have revised the "Operational Definitions" section (Lines 174–183) to address the conceptual concerns. Specifically, we have: Standardized the definition of Practice to "at least one dose" to ensure internal consistency.

Provided a technical justification for the 50% cut-off for knowledge and attitude, citing the modified Bloom’s criteria and relevant local literature [14].

Standardized the use of "good/poor" terminology across all KAP domains.

4. Data Availability – Not Compliant with Journal Policy

The statement “data available upon reasonable request” does not meet PLOS ONE requirements.

→ Authors must:

- Deposit data in a public repository, OR

- Provide a justified restriction (e.g., ethical/legal constraints with access mechanism).

Response- Thank you for this guidance regarding the PLOS ONE Data Availability Policy. We have updated our statement and our submission to ensure full compliance. We have now provided all relevant raw data as Supporting Information files.

These files include the fully de-identified datasets for both the Health Science and Social Science student cohorts, allowing for complete replication of our reported results, including the multivariable logistic regression and model diagnostics (VIF and Hosmer-Lemeshow tests).

5. Statistical Analysis – Insufficient Detail

The manuscript lacks key information:

- Variable selection criteria for multivariable models

- Multicollinearity assessment

- Model diagnostics (e.g., Hosmer-Lemeshow test)

Some reported findings are unstable (wide CIs), e.g.: AOR = 7.61 (1.65–35.12)

→ Interpret cautiously and acknowledge limited precision.

Response- We have comprehensively revised the statistical reporting to adhere to PLOS ONE’s standards: Variable Selection: We have specified that variables with p < 0.25 in bivariable analysis were entered into the multivariable model using backward stepwise elimination (Methods, Lines 215–226). Diagnostics: We now report Hosmer–Lemeshow test results, Nagelkerke R², and Classification Accuracy for all models. For the SSS knowledge model, we have justified its use despite the H-L result by highlighting its 80.3% predictive accuracy. Multicollinearity: We have confirmed model stability with VIF values for all predictors (HSS Max VIF = 1.836; SSS Max VIF = 1.252). Precision: We have added cautionary statements regarding variables with wide confidence intervals in both the Results and Limitations.

6. Results Section – Reporting Issues

In Table 2: Total respondents are incorrectly described as 335 instead of 670 in text.

- Source of information denominator (n=286) is unclear→ Explain missing data handling.

- Some tables: Lack clarity (e.g., Table 5 formatting, inconsistent totals), avoid redundancy between text and tables.

Response- We have corrected these reporting issues to ensure full transparency: Sample Size Consistency: We have updated the text and Table 2 to consistently reflect the total sample (N=670) and the equal distribution between the two study cohorts (n=335 each).

Denominator Clarification: We have clarified that the denominator for 'Source of Information' is derived only from participants with prior HPV awareness (n=470 total). We have added a specific footnote to Table 2 to explain this skip-logic, confirming that no data was missing, but rather that the question was only applicable to aware participants.

Table 5 Refinement: We have reformatted Table 5 for clarity and removed redundant descriptions from the text, as suggested.

7. Interpretation – Overstatement of Findings

The concept of “window of disengagement” is interesting but:

- Not directly measured

- Based on cross-sectional data → cannot infer temporal trends

Claims regarding: social media “misinformation” and Knowledge decay over time→ These are speculative and should be toned down.

Response- We have systematically toned down all causal and overreaching claims throughout the manuscript. First, we replaced causal language such as "knowledge decay" and "window of disengagement" with descriptive phrasing (e.g., "lower knowledge levels observed in later academic years") and added a disclaimer that these are hypotheses requiring longitudinal testing, as cross-sectional data cannot establish temporal trends. Second, we softened claims regarding Ethiopia's 90-70-90 targets: "requires" was changed to "could potentially contribute to," and we explicitly state that cross-sectional KAP studies cannot directly measure progress toward elimination targets. Third, we reframed speculation about misinformation as hypotheses (e.g., "may be associated with," "generates the hypothesis that") and added an explicit acknowledgment that we did not directly measure misinformation exposure. Finally, we added a new sentence to the Limitations section stating that all observed associations are correlational and causal inferences cannot be drawn. All changes are highlighted in the revised manuscript.

8. Discussion – Needs Better Balance

- The discussion is overly narrative and repetitive.

- Some comparisons lack critical appraisal of differences in study settings.

- Limited engagement with Ethiopian national HPV policy context, Vaccine availability and cost barriers

Response- We have revised the Discussion (Lines 406 to 418 and 426 to 429) to provide a rigorous contextualization of our vaccination rates using recent Ethiopian literature:

Regional Alignment: We noted that our results align with findings from Bahir Dar (16%), confirming the validity of our data in the Amhara region.

Target Group Disparity: We contrast our 18.2% rate with the 66.5% reported in primary schools (Kassa et al., 2021), explaining that university students fall into a "policy gap" as they are outside the primary target age for national school-based campaigns.

National Comparison: We have benchmarked our findings against the 2025 PMA national survey (30.8%) and the 2023 regional meta-analysis (41.46%), highlighting that university-aged women represent a underserved population in Ethiopia’s current vaccine rollout strategy.

Minor Comments

9. Language and Grammar

The manuscript requires extensive English editing:

- Capitalization errors (e.g., “Prevalent”, “Cervical Cancer”)

- Inconsistent tense usage

Suggest professional language editing.

Response- The manuscript has undergone extensive professional English editing. We have corrected capitalization errors and ensured consistent use of the past tense throughout the Methods and Results sections.

10. Abstract:

- Methods section is too dense

- Include clearer reporting of effect sizes with CI

Response- Abstract: The Methods section of the abstract has been simplified for better readability. All reported findings now include explicit Adjusted Odds Ratios (AOR) with their corresponding 95% Confidence Intervals (CI).

11. Terminology Consistency

- Ensure consistent use of:

“Health Science Students (HSS)”

“Social Science Students (SSS)”

Avoid switching terms throughout.

Response- We have standardized the terminology across the manuscript, consistently using "Health Science Students (HSS)" and "Social Science Students (SSS)."

12. Tables and Formatting

Improve:

- Table titles (more descriptive)

- Alignment and formatting

- Remove unnecessary duplication.

Response- Tables: Table titles have been revised to be more descriptive. We have improved the formatting and alignment of all tables and removed redundant data descriptions from the text.

13. References

Some references:

- Appear incomplete or inconsistently formatted

- Ensure adherence to journal style.

Response- : All references have been audited for completeness and reformatted to strictly adhere to the PLOS ONE (Vancouver) style.

Strengths of the Study

Large sample size (n=670

# Comparative design across disciplines

# Focus on an under-researched population in Ethiopia

# Policy-relevant findings aligned with global target

Recommendation

Major Revision

The manuscript has strong potential but requires substantial methodological clarification, correction of inconsistencies, and improved analytical rigor before it can be considered for publication.

Reviewer #2: The manuscript addresses an important public-health topic (HPV knowledge, attitude, and prevention practices among university students). However, address the issues to improve the article standard.

1. The manuscript states: “prospective institutional-based comparative cross-sectional study”

This is methodologically incorrect terminology. A cross-sectional study cannot be prospective unless repeated measurement or follow-up exists.

Response- We thank the reviewer for identifying this inconsistency. We have removed the term "prospective" from the Abstract and the Methods section. The study is now consistently described as a "comparative cross-sectional study" throughout the manuscript.

2. In line 42, 43: Author claimed However, both groups

43 exhibited poor knowledge (HSS: 61.2%; SSS: 80.9%). How it was measured? is more than 60% poor?

Response: We appreciate the opportunity to clarify this. The percentages reported (HSS: 61.2%; SSS: 80.9%) refer to the proportion of students in each group who were categorized as having poor knowledge.

Measurement Criteria: "Poor knowledge" was defined as achieving a score of less than 50% on the knowledge-based questions (modified Bloom’s cut-off). Therefore, 61.2% of Health Science students failed to correctly answer at least half of the knowledge questions. We have clarified this definition in the Operational Definitions section (Line 172 to 175) to prevent further ambiguity.

3. Outcome variables categorized as:good/poor, cut-off criteria should have reference.

Response: We agree with the reviewer’s suggestion. We have updated the Operational Definitions section to include the scientific justification for the categorization of our outcome variables. Specifically, the 50% cut-off point used for knowledge and attitude scores follows the modified Bloom’s criteria, a standard threshold in similar epidemiological studies conducted in Ethiopia. We have added the following reference to support this:

Mekonnen BA, Anagaw YK, Kassahun BA, Worku MC. Evaluation of female university students’ knowledge, attitudes, and practices toward human papillomavirus infection and vaccination. Multicenter cross-sectional study. BMC Women's Health. 2024;24(1):437.

These changes can be found in the revised manuscript in the Methods section (Lines 172–185).

4. The study states:

selected via multistage sampling However: Missing: • sampling frame • stage-wise selection steps • proportional allocation method • response rate

Without these: Risk of selection bias

Response: We have revised the sampling section (Lines 117–125) to provide full methodological transparency.

Design Clarification: We have clarified that a quota-based convenience sampling approach was used instead of a probability-based multistage design, as a formal sampling frame (student roster) was unavailable.

Stage-wise Selection: We now describe the two-stage process: purposive college selection followed by proportional allocation across departments.

Response Rate: We have explicitly stated the response rate (100%) and explained the data collection window (June to July 2025).

Selection Bias: To address the reviewer's concern, we have also added a statement in the Limitations section acknowledging the risk of selection bias associated with this non-probability sampling method.

5. Regression model includes predictors but manuscript does not explain: whether variables entered based on p<0.25/p<0.20/p<0.05 screening • stepwise vs forced entry model. Standard epidemiologic reporting missing.

Response- We have revised the Data Processing and Analysis section (Lines 215–224) to provide the missing epidemiological details. We now explicitly state that: A p-value threshold of < 0.25 in the bivariable analysis was used for initial variable screening.

A backward stepwise elimination method was employed for the multivariable model building to identify independent predictors while controlling for confounders.

6. Multicollinearity assessment not reported. Expected reporting:VIF values ,tolerance values, Hosmer–Lemeshow test, pseudo-R² missing.

Response- We have updated the manuscript to include comprehensive diagnostic reporting for all multivariable logistic regression models. These additions ensure the statistical validity and reliability of our findings:

Multicollinearity: We have now reported the Variance Inflation Factor (VIF) values to confirm the absence of significant collinearity. All VIF values were well below the threshold of 10 (HSS Max VIF = 1.836; SSS Max VIF = 1.252).

Model Fitness: We have included the results of the Hosmer–Lemeshow test for each model to demonstrate adequate goodness-of-fit.

Explanatory Power: We have added the Nagelkerke R-square ($Pseudo-R^2$) and overall classification accuracy (e.g., 80.3% for the SSS knowledge model) to provide a clearer picture of the models' predictive utility.

6. In table 5, Why 5th year was used as reference?. It should be 1st. In table 6, 0.00 was reported in the academic year. It should be 0.001*

Response- As requested, we have recalculated the associations in Table 5 using the 2nd Year as the reference category. This adjustment provides a more intuitive interpretation of how students' attitudes shift as they progress through their academic program. While the odds of having a good attitude were higher in the 5th year compared to the 2nd year (COR = 2.46), the result remained statistically non-significant (p = 0.06). We have updated Table 5 and the corresponding text (Lines [Insert Line #]) to reflect these new values.

7. Vaccination rate discussion insufficient: Vaccination rates extremely low:• 18.2% • 15.2%

But manuscript does not discuss: vaccine availability , • national rollout status • cost barriers or policy context.

Response - We thank the reviewer for this insightful comment. We have significantly expanded the Discussion section (Lines [Insert Line #s]) to provide a multi-layered analysis of the low vaccination rates (15.2%–18.2%):

Policy Context: We now discuss the findings within the framework of Ethiopia’s National Cancer Control Plan (2025–2029) and the WHO "90-70-90" global targets.

Target Group Mismatch: We have clarified that Ethiopia’s National Expanded Program on Immunization primarily targets 9–14-year-olds. University students represent a "missed cohort" who are not prioritized in routine campaigns.

Systemic Barriers: We have added a discussion on logistical hurdles, including inconsistent supply, cold-chain requirements, and the financial barriers faced by young adults who must access the vaccine outside of school-based programs.

National Rollout Status: We have updated the text to reflect Ethiopia’s recent transition to a single-dose schedule and how actual uptake in higher education settings lags behind national goals for younger cohorts.

8. Literature comparison limited

Discussion lacks:• regional comparison (Africa / Asia) • LMIC HPV vaccination studies • university-level interventions evidence.

Response- We have significantly expanded the Discussion section (Lines 426 to 436) to provide regional and global context. Specifically, we have added comparisons with study findings from Nigeria (Reference 26), India (Reference 27), and Malaysia (Reference 28). These additions highlight that the low vaccination uptake and "catch-up gap" observed among our students are consistent with challenges faced by university populations across other LMICs in Africa and Asia.

9. Over-interpretation risk; Study claims implications for: achieving Ethiopia’s 90-70-90 targets However: Cross-sectional KAP studies cannot evaluate progress toward elimination targets. This should be softened.

Response- We agree with the reviewer that a cross-sectional study has inherent limitations in evaluating progress toward long-term global targets. We have revised the language in the Discussion and Conclusion (Lines 448 to 453) to "soften" these claims. We now state that our findings inform the challenges and identify barriers to reaching the 90-70-90 targets, rather than directly evaluating Ethiopia's progress toward them.

Attachment Submitted filename: 1 Author respons.docx

# Peer review: Decision Letter 1

**DOI:** 10.1371/journal.pone.0359838.r003

**Authors:** Mengistu Zenebe

14 Aug 2026

Dear Dr. malkamu,

Thank you for submitting your manuscript to PLOS ONE. After careful consideration, we feel that it has merit but does not fully meet PLOS ONE’s publication criteria as it currently stands. Therefore, we invite you to submit a revised version of the manuscript that addresses the points raised during the review process.

Please submit your revised manuscript by Sep 28 2026 11:59PM. If you will need more time than this to complete your revisions, please reply to this message or contact the journal office at plosone@plos.org. When you're ready to submit your revision, log on to https://www.editorialmanager.com/pone/ and select the 'Submissions Needing Revision' folder to locate your manuscript file.

- A letter that responds to each point raised by the academic editor and reviewer(s). You should upload this letter as a separate file labeled 'Response to Reviewers'.
- A marked-up copy of your manuscript that highlights changes made to the original version. You should upload this as a separate file labeled 'Revised Manuscript with Track Changes'.
- An unmarked version of your revised paper without tracked changes. You should upload this as a separate file labeled 'Manuscript'.

If you would like to make changes to your financial disclosure, please include your updated statement in your cover letter. Guidelines for resubmitting your figure files are available below the reviewer comments at the end of this letter.

If applicable, we recommend that you deposit your laboratory protocols in protocols.io to enhance the reproducibility of your results. Protocols.io assigns your protocol its own identifier (DOI) so that it can be cited independently in the future. For instructions see: https://journals.plos.org/plosone/s/submission-guidelines#loc-laboratory-protocols. Additionally, PLOS ONE offers an option for publishing peer-reviewed Lab Protocol articles, which describe protocols hosted on protocols.io. Read more information on sharing protocols at https://plos.org/protocols?utm_medium=editorial-email&utm_source=authorletters&utm_campaign=protocols.

As the corresponding author, your ORCID iD is verified in the submission system and will appear in the published article. PLOS supports the use of ORCID, and we encourage all coauthors to register for an ORCID iD and use it as well. Please encourage your coauthors to verify their ORCID iD within the submission system before final acceptance, as unverified ORCID iDs will not appear in the published article. *Only*  the individual author can complete the verification step; PLOS staff *cannot*  verify ORCID iDs on behalf of authors.

We look forward to receiving your revised manuscript.

Kind regards,

Mengistu Hailemariam Zenebe, PhD

Academic Editor

PLOS One

**Journal Requirements:**

1. If the reviewer comments include a recommendation to cite specific previously published works, please review and evaluate these publications to determine whether they are relevant and should be cited. There is no requirement to cite these works unless the editor has indicated otherwise.

2. Please review your reference list to ensure that it is complete and correct. If you have cited papers that have been retracted, please include the rationale for doing so in the manuscript text, or remove these references and replace them with relevant current references. Any changes to the reference list should be mentioned in the rebuttal letter that accompanies your revised manuscript. If you need to cite a retracted article, indicate the article’s retracted status in the References list and also include a citation and full reference for the retraction notice.

[Note: HTML markup is below. Please do not edit.]

Reviewers' comments:

Reviewer's Responses to Questions

**Comments to the Author**

Reviewer #2: All comments have been addressed

Reviewer #3: (No Response)

Reviewer #4: All comments have been addressed

**********

2. Is the manuscript technically sound, and do the data support the conclusions?

Reviewer #2: Yes

Reviewer #3: Yes

Reviewer #4: Partly

**********

3. Has the statistical analysis been performed appropriately and rigorously?

Reviewer #2: Yes

Reviewer #3: Yes

Reviewer #4: Yes

**********

4. Have the authors made all data underlying the findings in their manuscript fully available?

The PLOS Data policy

Reviewer #2: Yes

Reviewer #3: Yes

Reviewer #4: Yes

**********

5. Is the manuscript presented in an intelligible fashion and written in standard English?

Reviewer #2: Yes

Reviewer #3: Yes

Reviewer #4: Yes

**********

**Reviewer #2:**  (No Response)

**Reviewer #3:**  Manuscript: Knowledge, Attitudes, and Prevention Practices Regarding Human Papillomavirus Among Health and Social Science Female Students in Northwest Ethiopia: A Comparative Cross-Sectional Study

Manuscript ID: PONE-D-26-09491R1

Overall Assessment

This manuscript addresses an important public health issue by comparing HPV-related knowledge, attitudes, and prevention practices among female health science and social science students. The relatively large sample, inclusion of two academic groups, and focus on HPV vaccination and cervical cancer prevention are notable strengths. The findings are relevant for university health education and may contribute useful evidence from Northwest Ethiopia. The manuscript is generally suitable for publication after minor revisions aimed primarily at improving clarity, consistency, and reporting.

Title and Abstract

The title is clear and appropriately reflects the study design and population. Minor editing is needed for capitalization and consistency, particularly “comparative cross-sectional study.” In the abstract, the word “prospective” should be removed because the study is cross-sectional. The authors should also report the response rate and ensure that all abbreviations, including HSS and SSS, are defined at first use. The conclusion should remain closely aligned with the observational findings and avoid implying that the proposed educational interventions were tested.

Introduction

The introduction provides an adequate overview of HPV, cervical cancer prevention, vaccination, and the importance of university students as a target population. The use of the Health Belief Model strengthens the conceptual foundation. However, the authors should briefly clarify how the model informed questionnaire development or interpretation. The term “multicentre” should be replaced because the study was conducted at one university. The final paragraph would benefit from a concise statement of the primary comparative objective and the expected difference between the two academic groups.

Methods

The study design, setting, sample, and data-collection period are generally described clearly. Minor clarification is required regarding the sampling procedure because both simple random sampling and convenience sampling are mentioned. The authors should revise this section to provide one consistent account of how participants were selected within each college and department.

The questionnaire description should include the number of items in each knowledge, attitude, and practice domain, the possible score ranges, and any reliability coefficients obtained during pretesting. The 50% cutoff used to define good and poor knowledge and attitude should be supported with a reference or brief justification.

The definition of “practice” is based primarily on completion of the two-dose HPV vaccination schedule. The authors should therefore consider using the more precise term “vaccination practice” or “HPV vaccine uptake” throughout the manuscript. The wording regarding inter-rater reliability should also be clarified because the questionnaire was self-administered.

The ethics information must be harmonized. The manuscript and submission form report different protocol numbers and approval dates. The correct approval details should be verified and presented consistently.

Results

The results are informative and clearly demonstrate higher HPV awareness and more positive attitudes among health science students, while vaccination completion remained low in both groups. Tables are generally understandable, but several minor revisions are needed.

The authors should add the relevant chi-square test results and exact p values for comparisons between health and social science students. In the regression tables, p = 0.00 should be reported as p < .001. Exact p values should be provided for borderline findings rather than using p = .05. Wide confidence intervals should be acknowledged as indicating imprecision.

The denominators used for the sources-of-information percentages in Table 2 should be checked and clearly stated for each academic group. Table titles should also accurately refer to both health and social science students where both groups are presented.

Discussion

The discussion appropriately interprets the greater awareness and more positive attitudes among health science students and the persistently low vaccination uptake in both groups. Comparison with previous Ethiopian and international studies is relevant. The authors should slightly reduce repetition and clearly distinguish awareness, knowledge, attitudes, and vaccination practice.

Explanations related to curriculum exposure, social media use, access, and vaccine availability should be framed as possible interpretations rather than confirmed causes. The implications section may retain recommendations for discipline-specific education, but these should be presented as recommendations arising from the findings rather than proven interventions.

The limitations section should briefly acknowledge the single-university setting, cross-sectional design, self-reported data, possible recall bias, and limited generalizability.

Recommendation

Minor revision. The manuscript is relevant, adequately designed, and potentially publishable. The required revisions are mainly related to reporting consistency, clarification of sampling and measurement procedures, correction of ethics details, improved statistical presentation, data-availability compliance, and language editing.

**Reviewer #4:**  Re-Review Report

Manuscript Title: Knowledge, Attitudes, and Prevention Practices Regarding Human Papillomavirus Among Health and Social Science Female Students in Northwest Ethiopia: A Comparative Cross-sectional Study

Manuscript Number: PONE-D-26-09491R1

1. Overall Assessment

The authors have provided a comprehensive and diligent response to the reviewers' and Academic Editor's comments. The revised manuscript demonstrates substantial improvements in methodological transparency, statistical rigor, and interpretive caution. The authors have addressed nearly all major concerns raised, including correcting the study design terminology, resolving sampling inconsistencies, standardizing outcome definitions, and providing robust statistical diagnostics.

However, several critical issues remain unresolved in the manuscript text itself, despite being acknowledged in the response letter. These issues pertain to sampling methodology, data availability, and table formatting. While the authors have stated their intention to make these corrections, the revised manuscript file does not consistently reflect these changes. Therefore, I recommend Major Revision to allow the authors to ensure that all promised revisions are fully implemented in the manuscript text.

2. Evaluation of Response to Specific Criticisms

The authors' response to the reviewers' comments was thorough and addressed most points substantively. The following is a summary of key improvements and remaining issues:

Addressed Issues

Issue Resolution

Study Design Terminology Removed "prospective" throughout; now consistently describes as "comparative cross-sectional"

Health Belief Model Removed theoretical framing as HBM constructs were not operationalized

Outcome Definitions Standardized "practice" as "at least one dose"; provided justification for 50% cut-off citing modified Bloom's criteria and Mekonnen et al. 2024

Statistical Reporting Added variable selection criteria (p<0.25), backward stepwise elimination, VIF values, Hosmer-Lemeshow test results, Nagelkerke R², and classification accuracy

Interpretation Toned down causal language; replaced "knowledge decay" and "window of disengagement" with descriptive phrasing; softened claims regarding 90-70-90 targets

Discussion Context Expanded with regional comparisons (Nigeria, India, Malaysia), policy context, and Ethiopian national data (PMA 2025, meta-analysis 2023)

Language Manuscript has undergone professional English editing

References Reformatted to PLOS ONE (Vancouver) style

3. Critical Unresolved Issues

Issue 1: Sampling Methodology - Inconsistent Implementation

Problem: The authors' response letter states they have revised the sampling section to reflect quota-based convenience sampling. However, the revised manuscript text (Lines 117-125 in the PDF) contains both the corrected text AND remnants of the original multi-stage sampling description. Specifically:

• The Methods section still contains the sentence: "The study utilized a multi-stage sampling procedure. First, the College of Health Sciences and the College of Social Sciences and Humanities were purposively selected... Second... selected using a simple random sampling technique (SRS)."

• This directly contradicts the authors' stated revision to use quota-based convenience sampling.

Required Action: The authors must remove all remaining references to multi-stage and random sampling from the manuscript text. The sampling description should be clean and consistent, describing only the quota-based convenience sampling approach with the two-stage process (purposive college selection followed by proportional allocation across departments). The Limitations section must explicitly acknowledge selection bias associated with this non-probability sampling method.

Issue 2: Data Availability Statement - Not Compliant

Problem: The Data Availability Statement in the manuscript (Line 503) still reads: "Data available on request from the authors." This does not comply with PLOS ONE's policy requiring data to be fully available without restriction.

Response Letter Claim: The authors state they have "provided all relevant raw data as Supporting Information files" (S1 Data and S2 Data).

Required Action: The Data Availability Statement in the manuscript must be updated to clearly state that all relevant data are included as Supporting Information files, not "available on request." The current text should be replaced with something like: "All relevant data supporting the conclusions of this study are included as Supporting Information files (S1 Data: Health Science Students and S2 Data: Social Science Students)."

Issue 3: Table Formatting and Clarity Issues

Problem: Several tables in the revised manuscript remain poorly formatted and difficult to interpret:

Table Issue

Table 4 Formatting is inconsistent; some cells contain merged text (e.g., "04.7704" appears to be a formatting error for "0.7704"); confidence intervals are unclear or malformed

Table 5 The reference category was changed from 5th year to 2nd year in the response, but the table text still contains confusing formatting with multiple OR values and inconsistent p-values

Table 6 Contains "0.3504*" with no corresponding footnote explaining the asterisk; "13.5039" appears as a malformed OR value; some cells contain merged text (e.g., "0.9043 (0.4803-19.68+)")

Table 7 Formatting is acceptable but some OR values appear unusual (e.g., "0.2798 (0.7784-24.105)" - OR < 1 but CI > 1 suggests potential data issues)

Required Action: All tables must be carefully reformatted for clarity and consistency. Specific requirements:

a) Ensure all OR/AOR values are reported with clear confidence intervals (e.g., AOR = X.XX, 95% CI: Y.YY-Z.ZZ)

b) Correct malformed numerical entries (e.g., "04.7704" should be "0.7704")

c) Add footnotes explaining all abbreviations and asterisks

d) Ensure reference categories are clearly indicated and consistent

e) Use consistent decimal places throughout

Issue 4: Incomplete Resolution of "Window of Disengagement" Terminology

Problem: The authors state in their response that they replaced "window of disengagement" with descriptive phrasing. However, the term still appears in the Discussion (Line 386): "the observed gap in later academic years window of disengagement persists" - this appears to be an incomplete edit where the original phrase was not fully removed.

Required Action: Remove the phrase "window of disengagement" completely from the manuscript text. Use descriptive phrasing throughout, such as "lower knowledge levels observed in later academic years" or "observed gaps in later academic years."

Issue 5: Inconsistent p-value Reporting

Problem: The authors acknowledged the need to correct p-values reported as "0.00" to "<0.001." However, Table 6 still contains "0.00*" in the knowledge model for the 2nd year category (p<0.001). Additionally, some p-values in tables still show "0.000" formatting.

Required Action: Ensure all p-values are reported as "<0.001" (not "0.00" or "0.000") throughout all tables and text.

Issue 6: Incorrect Reference Category Justification

Problem: The authors changed the reference category in Table 5 from 5th year to 2nd year but did not provide a clear rationale for this choice in the manuscript text. The use of 2nd year as reference is more interpretable, but this should be explicitly stated.

Required Action: Add a brief explanation in the Statistical Analysis section or table footnote justifying the choice of reference category (e.g., "2nd year was used as the reference category to provide a more intuitive interpretation of changes in attitudes as students’ progress through their academic program").

4. Minor Issues (Optional but Recommended)

Issue Recommendation

Duplicate Text in Abstract The abstract contains a duplicate Methods section (Lines 35-38 and Lines 39-41 appear to be repeated with slight variations). Remove the duplicate.

Inconsistent Use of "Social Science Students" The manuscript sometimes uses "Social Science" and other times "social science" - standardize capitalization throughout.

Table 2 Footnote Clarity The note states: "The percentages for 'Source of Information' are calculated based on the subset of participants who reported prior awareness" - this is clear, but consider adding the actual denominator values (n=280 for HSS, n=190 for SSS) to the table or footnote for transparency.

Reference 14 Citation The authors cite Mekonnen et al. 2024 for the 50% cut-off justification. Ensure this reference is correctly formatted and appears in the reference list.

5. Summary Table of Required Revisions

Priority Issue Location Required Action

Critical Sampling methodology inconsistency Methods: Sampling section Remove all remaining references to multi-stage and random sampling; ensure only quota-based convenience sampling is described

Critical Data Availability Statement End of manuscript Update statement to indicate data are included as Supporting Information files

Critical Table formatting (Table 4, 5, 6) Results section Correct malformed numerical entries; ensure consistent formatting of OR/AOR and CIs; add footnotes

Critical "Window of disengagement" term Discussion (Line 386) Remove the phrase completely and use descriptive phrasing

Critical p-value reporting Tables 4, 6 Change "0.00" to "<0.001"

Minor Duplicate text in Abstract Abstract Remove duplicate Methods paragraphs

Minor Terminology consistency Throughout Standardize capitalization of "Social Science"

Minor Reference category rationale Methods/Table 5 Add brief explanation for using 2nd year as reference

**********

what does this mean?). If published, this will include your full peer review and any attached files.

If you choose “no”, your identity will remain anonymous but your review may still be made public.

**Do you want your identity to be public for this peer review?**  For information about this choice, including consent withdrawal, please see our Privacy Policy

Reviewer #2: No

Reviewer #3: No

Reviewer #4: **Yes:**  Mohammed Abu Sayeed, Associate Professor, Department of Pharmacy, International Islamic University Chittagong, Bangladesh

**********

[NOTE: If reviewer comments were submitted as an attachment file, they will be attached to this email and accessible via the submission site. Please log into your account, locate the manuscript record, and check for the action link "View Attachments". If this link does not appear, there are no attachment files.]

To ensure your figures meet our technical requirements, please review our figure guidelines: https://journals.plos.org/plosone/s/figures

You may also use PLOS’s free figure tool, NAAS, to help you prepare publication quality figures: https://journals.plos.org/plosone/s/figures#loc-tools-for-figure-preparation.

NAAS will assess whether your figures meet our technical requirements by comparing each figure against our figure specifications.

# Author comment: Author response to Decision Letter 2

**DOI:** 10.1371/journal.pone.0359838.r004

25 Aug 2026

Author response

The authors express their sincere gratitude to the editor and reviewers for their insightful feedback and dedication to elevating the quality of this manuscript. Point-by-point responses to each of the editor's and reviewers' comments are detailed below, along with the corresponding revisions made to the manuscript.

Reviewer 3 comments and Author responses

1. Minor editing is needed for capitalization and consistency, particularly 'comparative cross‑sectional study.

Author response: We have corrected the capitalization in the title to read "Comparative Cross‑Sectional Study" as suggested.

2. In the abstract, the word 'prospective' should be removed because the study is cross‑sectional.

Author response: We have reviewed the abstract and the entire manuscript and confirm that the word "prospective" does not appear anywhere. The study design is consistently described as "cross‑sectional" throughout. No changes were needed for this comment.

3. Ensure that all abbreviations, including HSS and SSS, are defined at first use.

Author Response: We confirm that HSS (Health Science Students) and SSS (Social Science Students) are already defined at first use in the abstract (lines 42 and 43). No changes were required.

4. The conclusion should remain closely aligned with the observational findings and avoid implying that the proposed educational interventions were tested.

Author response: We have revised the conclusion to more clearly frame recommendations as implications of the observational findings, rather than implying that interventions were tested. The revised text now states: "Based on our observational findings, we recommend..."

5. Briefly clarify how the model informed questionnaire development or interpretation.

Author response: We have clarified in the Introduction (lines 77‑78) that the study was guided by a comparative empirical framework and did not impose a specific behavioral theory. The HBM was therefore not used to inform questionnaire development.

6. The term 'multicentre' should be replaced because the study was conducted at one university."

Author response: We have replaced the term "multicenter" with "comparative" in line 86, as the study was conducted at a single university (Debre Tabor University).

7. The final paragraph would benefit from a concise statement of the primary comparative objective and the expected difference between the two academic groups.

Author response: We have added a concise statement of the expected difference between groups to the final paragraph of the Introduction (lines 90‑92), stating that we hypothesized Health Science students would have higher knowledge and more favorable attitudes due to curriculum exposure.

8. Clarification is required regarding the sampling procedure because both simple random sampling and convenience sampling are mentioned.

Author response: We confirm that the sampling procedure is consistently described as "non‑probability quota‑based convenience sampling" and "purposive selection" of colleges. No mention of simple random sampling appears in the manuscript. No changes were required.

9. The questionnaire description should include the number of items in each domain, the possible score ranges, and reliability coefficients.

Author response: We thank the reviewer for this valuable suggestion. We have expanded the questionnaire description in the Methods section to specify the number of items and possible score ranges for each domain. Additionally, we have now calculated and reported Cronbach's alpha coefficients to assess internal consistency for both Health Science and Social Science student cohorts.

For the knowledge domain (14 items), Cronbach's alpha was 0.930 in both Health Science and Social Science students, indicating excellent internal consistency. For the attitude domain (5 items), Cronbach's alpha was 0.762 in both groups, indicating acceptable reliability. Corrected item-total correlations for all items exceeded 0.30, confirming that each item contributed meaningfully to its respective scale.

As the practice domain was measured as a single binary outcome (vaccination status: yes/no), Cronbach's alpha was not applicable for that domain. These reliability statistics have been added to the Data Quality Control section of the manuscript.

10. The 50% cut‑off used to define good and poor knowledge and attitude should be supported with a reference or brief justification.

Author response: We have expanded the justification for the 50% cut‑off in the Operational Definitions section (lines 154‑155) to reference modified Bloom's criteria and prior Ethiopian studies.

11. The definition of 'practice' is based primarily on completion of the HPV vaccination schedule. Consider using the more precise term 'vaccination practice' or 'HPV vaccine uptake'.

Author response: We have revised the terminology throughout the manuscript to use "vaccination practice" or "HPV vaccine uptake" where appropriate, and have updated Table 3 and the Abstract accordingly.

12. The wording regarding inter‑rater reliability should also be clarified because the questionnaire was self‑administered.

Author response: We have clarified that the inter‑rater reliability checks were conducted on the data abstraction process, not on the self‑administered questionnaire itself (lines 179).

13. The manuscript and submission form report different protocol numbers and approval dates. Verify and present consistently.

Author response: We have verified the ethics protocol number and approval date against the official approval letter. The manuscript now consistently reports protocol number 017/2024 (July 20, 2024), matching the submission form.

14. Add the relevant chi‑square test results and exact p‑values for comparisons between health and social science students.

Author response: We thank the reviewer for this valuable suggestion. To address this, we created a grouping variable (academic_discipline) in both datasets before merging (1 = Health Science, 2 = Social Science). Using the combined dataset (N=670), we performed Pearson's chi-square tests to formally compare the two groups across all key outcomes. The results, now added to the Results section (lines 240-245), show:

HPV knowledge (good knowledge): χ² = 31.61, df = 1, p < 0.001

Attitude towards HPV vaccination (good attitude): χ² = 26.07, df = 1, p < 0.001

Vaccination practice (good practice): χ² = 1.07, df = 1, p = 0.300

These formal comparisons confirm that Health Science students have significantly higher knowledge and more favorable attitudes compared to Social Science students, while vaccination practice does not differ significantly between the two groups. The non-significant practice finding aligns with the low overall vaccination rates observed in both groups (18.2% vs. 15.2%).

15. In the regression tables, p = 0.00 should be reported as p < .001.

Author Response: All p‑values previously reported as "0.00" have been corrected to "<0.001" in accordance with standard reporting guidelines.

16. Wide confidence intervals should be acknowledged as indicating imprecision.

Author Response: We have already acknowledged the wide confidence intervals in the Results section (lines 257‑260) and noted that these estimates should be interpreted with caution. No additional changes were required.

17. The denominators used for the sources‑of‑information percentages in Table 2 should be checked and clearly stated for each academic group.

Author Response: We confirm that the denominators for the sources‑of‑information percentages in Table 2 are already clearly stated in the footnote (n=280 for HSS and n=190 for SSS). No changes were required.

18. Clearly distinguish awareness, knowledge, attitudes, and vaccination practice.

Author Response: We confirm that awareness, knowledge, attitudes, and vaccination practice are already clearly defined and distinguished in the Operational Definitions section (lines 150‑162) and are used consistently throughout the manuscript. No changes were required.

19. Explanations related to curriculum exposure, social media use, access, and vaccine availability should be framed as possible interpretations rather than confirmed causes.

Author Response: We have reviewed the Discussion and replaced definitive causal language with more cautious phrasing, using qualifiers such as "may," "likely," and "suggests" to frame explanations as possible interpretations rather than confirmed causes.

Reviewer 4 comments and author responses.

1. The Methods section contains remnants of the original multi‑stage sampling description. Remove all remaining references to multi‑stage and random sampling.

Author Response: We have reviewed the Sampling section and confirm that it consistently describes "non‑probability quota‑based convenience sampling" and "purposive selection" of colleges. No references to multi‑stage or random sampling remain in the manuscript. No changes were required for this comment.

2. The Data Availability Statement is not compliant with PLOS ONE policy. Update to indicate data are included as Supporting Information files.

Author Response: We confirm that the Data Availability Statement already states that all relevant data are included as Supporting Information files (Line 528-530 - All relevant data supporting the conclusions of this study are included as Supporting Information files (S1 Data: Health Science Students and S2 Data: Social Science Students), in compliance with PLOS ONE policy. No changes were required.

3. Tables 4, 5, and 6 are poorly formatted with malformed numerical entries. Correct all

Author Response: We have carefully reviewed Tables 4, 5, and 6 and corrected any formatting inconsistencies. All Adjusted Odds Ratios are now reported to two decimal places, confidence intervals are formatted consistently, and p‑values are presented as <0.001 where appropriate. Footnotes explaining abbreviations have been added.

4. The phrase 'window of disengagement' still appears in the Discussion. Remove it completely.

Author Response: We have removed the phrase "window of disengagement" from the manuscript (line 94) and replaced it with descriptive wording. We have also corrected the duplicate phrase in line 366 to "creating an observed gap in later academic years.

5. Table 6 still contains '0.00' – change to '<0.001'. Also ensure all p‑values are consistent.

Author Response: We have reviewed all regression tables and confirmed that p‑values are now consistently reported as <0.001 where appropriate. No instances of 0.00 or 0.000 remain.

6. Add a brief explanation for using 2nd year as the reference category in Table 5.

Author Response: We have added a brief explanation in the Statistical Analysis section (lines 284‑286) justifying the use of 2nd year as the reference category for academic year in Table 5. "For multivariable logistic regression, the reference category for academic year was set to 2nd year to provide a more intuitive interpretation of changes in attitudes and practices as students’ progress through their academic program."

7. The abstract contains a duplicate Methods section. Remove the duplicate.

Author response: We have reviewed the Abstract and confirm that there is no duplicate Methods section. The abstract follows a standard structure (Background, Objective, Methods, Results, Conclusion). No changes were required.

8. Standardize capitalization of 'Social Science' throughout.

Author response: We have standardized the capitalization of "Social Science" throughout the manuscript to ensure consistency.

9. Add actual denominator values (n=280 for HSS, n=190 for SSS) to Table 2 footnote.

Author response: We confirm that the denominators (n=280 for HSS and n=190 for SSS) are already clearly stated in the Table 2 footnote (Note: The percentages for 'Source of Information' are calculated based on the subset of participants who reported prior awareness of HPV (n=280 for Health Science and n=190 for Social Science). This explains the variation in denominators compared to the total sample size (N=670). No changes were required.

Attachment Submitted filename: 2_Author response.docx

# Peer review: Decision Letter 2

**DOI:** 10.1371/journal.pone.0359838.r005

**Authors:** Mengistu Zenebe

21 Sep 2026

<p>Knowledge, Attitudes, and Prevention Practices Regarding Human Papillomavirus Among Health and Social Science Female Students in Northwest Ethiopia: A Comparative cross-sectional Study

PONE-D-26-09491R2

Dear Author,

We’re pleased to inform you that your manuscript has been judged scientifically suitable for publication and will be formally accepted for publication once it meets all outstanding technical requirements.

Within one week, you’ll receive an e-mail detailing the required amendments. When these have been addressed, you’ll receive a formal acceptance letter and your manuscript will be scheduled for publication.

An invoice will be generated when your article is formally accepted. Please note, if your institution has a publishing partnership with PLOS and your article meets the relevant criteria, all or part of your publication costs will be covered. Please make sure your user information is up-to-date by logging into Editorial Manager at Editorial Manager® and clicking the ‘Update My Information' link at the top of the page. For questions related to billing, please contact billing support.

If your institution or institutions have a press office, please notify them about your upcoming paper to help maximize its impact. If they’ll be preparing press materials, please inform our press team as soon as possible -- no later than 48 hours after receiving the formal acceptance. Your manuscript will remain under strict press embargo until 2 pm Eastern Time on the date of publication. For more information, please contact onepress@plos.org.

Kind regards,

Mengistu Hailemariam Zenebe, PhD

Academic Editor

PLOS One

Additional Editor Comments (optional):

Reviewers' comments:

Reviewer's Responses to Questions

**Comments to the Author**

Reviewer #2: (No Response)

Reviewer #3: All comments have been addressed

**********

2. Is the manuscript technically sound, and do the data support the conclusions?

Reviewer #2: Partly

Reviewer #3: Yes

**********

3. Has the statistical analysis been performed appropriately and rigorously?

Reviewer #2: Yes

Reviewer #3: No

**********

4. Have the authors made all data underlying the findings in their manuscript fully available?

The PLOS Data policy

Reviewer #2: Yes

Reviewer #3: Yes

**********

5. Is the manuscript presented in an intelligible fashion and written in standard English?

Reviewer #2: Yes

Reviewer #3: Yes

**********

Reviewer #2: 1. The reference category for academic year is inconsistent between Table 5 and the accompanying note. The table appears to use fifth year as the reference category, whereas the note states that second year was used as the reference. Please verify the regression coding and revise the table, text, and footnote to ensure complete consistency.

2. Several regression estimates, particularly for prior HPV awareness and academic department, have wide confidence intervals. The authors should report the underlying cell frequencies and assess whether sparse data or separation may have contributed to the instability of the estimates.

3. In table 6, Heard about HPV p value was unnecessarily reported as bold format

Reviewer #3: The authors have satisfactorily addressed the reviewers’ comments and substantially improved the manuscript’s methodological clarity, statistical reporting, presentation of results, and interpretation of findings. The revisions are appropriate, and the manuscript is now suitable for publication. I have no further substantive concerns and recommend acceptance in its present form.

**********

what does this mean?). If published, this will include your full peer review and any attached files.

If you choose “no”, your identity will remain anonymous but your review may still be made public.

**Do you want your identity to be public for this peer review?**  For information about this choice, including consent withdrawal, please see our Privacy Policy

Reviewer #2: **Yes:**  Mohammad Injamul Hoq

Reviewer #3: No

**********

Attachment Submitted filename: PLOS_Review.pdf

# Editor report: Acceptance letter

**DOI:** 10.1371/journal.pone.0359838.r006

**Authors:** Mengistu Zenebe

PONE-D-26-09491R2

PLOS One

Dear Dr. malkamu,

I'm pleased to inform you that your manuscript has been deemed suitable for publication in PLOS One. Congratulations! Your manuscript is now being handed over to our production team.

At this stage, our production department will prepare your paper for publication. This includes ensuring the following:

* All references, tables, and figures are properly cited

* All relevant supporting information is included in the manuscript submission,

* There are no issues that prevent the paper from being properly typeset

You will receive further instructions from the production team, including instructions on how to review your proof when it is ready. Please keep in mind that we are working through a large volume of accepted articles, so please give us a few days to review your paper and let you know the next and final steps.

Lastly, if your institution or institutions have a press office, please let them know about your upcoming paper now to help maximize its impact. If they'll be preparing press materials, please inform our press team within the next 48 hours. Your manuscript will remain under strict press embargo until 2 pm Eastern Time on the date of publication. For more information, please contact onepress@plos.org.

You will receive an invoice from PLOS for your publication fee after your manuscript has reached the completed accept phase. If you receive an email requesting payment before acceptance or for any other service, this may be a phishing scheme. Learn how to identify phishing emails and protect your accounts at https://explore.plos.org/phishing.

If we can help with anything else, please email us at customercare@plos.org.

Thank you for submitting your work to PLOS One and supporting open access.

Kind regards,

PLOS One Editorial Office Staff

on behalf of

Dr. Mengistu Hailemariam Zenebe

Academic Editor

PLOS One
